# In‐Situ Vertical‐Contact Engineering of Laser‐Induced Graphene Nanotips for Ultra‐Sensitive Humidity Sensors

**DOI:** 10.1002/smll.202505017

**Published:** 2025-07-04

**Authors:** Ki Wan Kim, Won Gyun Park, Do‐Yeon Lee, Ga‐Won Lee, Binghao Wang, Jae‐Hyuk Ahn

**Affiliations:** ^1^ Department of Electronics Engineering Chungnam National University Daejeon 34134 Republic of Korea; ^2^ School of Electronic Science and Engineering Southeast University Nanjing Jiangsu 211189 China

**Keywords:** field ionization, humidity sensor, laser‐induced graphene, nanotips, respiratory monitoring, vertical contact

## Abstract

Advancements in laser‐induced graphene (LIG) technology enables the streamlined fabrication of 3D porous graphene‐based humidity sensors. However, conventional LIG‐based humidity sensors employing lateral‐contact configurations often exhibit limited responsivity, owing to their partially exposed structures and dominant subsurface current pathways. This study presents a novel vertical‐contact architecture that utilizes vertically aligned LIG nanotips for ultra‐sensitive humidity detection. Single‐pulse laser irradiation induces localized growth of the LIG nanotips, simultaneously forming in‐situ vertical contacts with both the top and bottom graphene electrodes. This fully exposed structure provides a highly efficient sensing interface. By adjusting the air‐gap height between the electrodes, the sensor operates in two distinct modes: Contact Mode, which achieves high responsivity (40%) via resistance modulation by water adsorption, and Remote Mode, which leverages field ionization to achieve exceptional responsivity (14 000%). The sensors demonstrate rapid response and recovery times (<1 s), excellent stability, and high gas selectivity. Integration into wearable face masks enables real‐time respiratory monitoring, including hyperventilation and high‐frequency breathing (up to 156 bpm), without signal degradation. This study presents a simple and scalable strategy for fabricating high‐performance humidity sensors for next‐generation wearable healthcare applications.

## Graphical abstract

This study presents a vertical‐contact architecture using fully exposed, vertically aligned laser‐induced graphene (LIG) nanotips for ultra‐sensitive humidity sensing. By leveraging sharp LIG nanotips for either direct conduction or noncontact field ionization, the sensor achieves over 100‐fold higher sensitivity than conventional designs and enables real‐time respiratory monitoring with fast response and excellent stability.

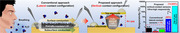

## 1 Introduction

Humidity‐sensing technology plays a vital role across diverse fields, including environmental monitoring, precision agriculture, pharmaceuticals, food safety, electronics manufacturing, and respiratory health monitoring.^[^ ^1^, ^2^, ^3^ ^]^ In particular, tracking humidity fluctuations during inhalation and exhalation is critical for respiratory diagnostics,^[^ ^4^, ^5^, ^6^, ^7^ ^]^ with established utility in monitoring exercise, sleep apnea,^[^ ^8^, ^9^ ^]^ respiratory diseases (such as asthma),^[^ ^10^ ^]^ and cardiovascular health conditions.^[^ ^11^ ^]^ Furthermore, this technology enables the detection of abnormal breathing patterns associated with psychological stress and anxiety disorders.^[^ ^12^ ^]^ For instance, mental stress triggers a physiological response characterized by an increased respiration rate, aimed at rapidly supplying energy and oxygen to the body.^[^ ^13^ ^]^

Transducing these biological signals into electrical outputs requires materials that can sensitively respond to external stimuli—such as moisture adsorption—by inducing measurable changes in current or resistance. Various materials, including polymers,^[^ ^14^, ^15^ ^]^ paper‐based substrates,^[^ ^16^, ^17^ ^]^ ceramics,^[^ ^18^ ^]^ 2D materials,^[^ ^19^, ^20^, ^21^ ^]^ and carbon‐based materials,^[^ ^22^, ^23^, ^24^ ^]^ have been explored to enhance humidity sensor performance. Among them, graphene has been extensively studied for humidity and gas sensing due to its excellent electrical conductivity, mechanical robustness, and high surface area.^[^ ^25^, ^26^, ^27^, ^28^, ^29^ ^]^ These intrinsic properties allow graphene to exhibit significant changes in its electrical characteristics upon interaction with water molecules, making it a highly effective material for humidity‐sensing applications.

Recently, 3D graphene structures have attracted considerable attention for gas and humidity sensors due to their larger surface area and enhanced porosity compared to conventional 2D counterparts.^[^ ^30^, ^31^, ^32^ ^]^ The increased number of active sites in 3D graphene facilitates more efficient molecular interactions, significantly improving sensing capabilities. Among the various fabrication methods, laser‐induced graphene (LIG) offers a simple and scalable approach to producing 3D porous graphene structures by locally carbonizing polyimide (PI) films with laser irradiation.^[^ ^33^ ^]^ Compared with traditional methods such as chemical vapor deposition (CVD) and epitaxial growth,^[^ ^34^, ^35^, ^36^ ^]^ LIG is more cost‐effective, enables room‐temperature processing, and does not require vacuum environments.^[^ ^37^, ^38^ ^]^ Additionally, the morphology and electrical properties of LIG can be precisely controlled by optimizing laser parameters; hence, tailored sensor performance can be realized.^[^ ^39^, ^40^ ^]^ Moreover, the applicability of LIG to various substrates—including natural materials such as paper, cork and wood—has broadened its usability, and the variations in porosity and surface composition among these substrates can enhance sensor performance.^[^ ^41^, ^42^, ^43^, ^44^ ^]^

However, developing highly sensitive LIG‐based humidity sensors for respiratory monitoring remains challenging. In conventional methods, LIG patterns are typically fabricated by scribing a PI substrate with a carbon dioxide (CO_2_) laser to form lateral‐contact configurations that detect current or resistance changes induced by humidity variations. Although LIG structures grow vertically from the substrate, forming a 3D porous network, only the top surface is directly exposed to ambient air. Consequently, interactions with water molecules are confined to the upper regions, whereas the lower embedded regions of the LIG remain inactive during the sensing process. This partially exposed structure fundamentally restricts the overall responsivity of the sensors. Owing to this structural constraint, conventional LIG‐based humidity sensors have struggled to achieve the ultra‐high responsivity required for advanced applications, such as real‐time respiratory monitoring.

To overcome the limitations of conventional LIG‐based humidity sensors, we propose a novel vertical‐contact architecture that enables a fully exposed sensing structure for ultra‐sensitive humidity detection. In this design, the graphene‐coated top and bottom electrodes were positioned parallel to each other with a defined air gap. Single‐pulse laser irradiation was employed to induce localized growth of vertically aligned LIG nanotips, which simultaneously formed in‐situ vertical contacts with both electrodes. This configuration resulted in a highly efficient and fully exposed 3D sensing interface.

By adjusting the air‐gap height, the sensor operates in two distinct modes: 1) Contact Mode, where the LIG nanotips directly contact the top electrode, forming narrow conductive pathways that are fully exposed to ambient moisture. The Contact Mode achieves a responsivity of 40% across a relative humidity (RH) range of 20%–90%; and 2) Remote Mode, in which the nanotips are spatially separated from the top electrode, enabling noncontact humidity sensing through field ionization. In the Remote Mode, the sharp curvature of the LIG nanotips generates intense localized electric fields, initiating an impact ionization process that exponentially amplifies the current response, resulting in an exceptional responsivity of 14 000%.

This dual‐mode operation provides both high responsivity and operational versatility, and the underlying mechanisms are validated through mathematical modeling and finite‐element simulations. Additionally, the proposed sensor demonstrated excellent long‐term stability, temperature stability, and high gas selectivity. To demonstrate practical applicability, we further integrated the sensors into wearable face masks, enabling real‐time respiratory monitoring with rapid response and recovery times (<1 s), even under dynamic conditions, such as hyperventilation and intense physical activity. These findings present new possibilities for developing advanced wearable healthcare monitoring systems.

## 2 Results and Discussion

### 2.1 In‐Situ Vertical‐Contact Engineering for Fully Exposed LIG Nanotips

**Figure 1:**
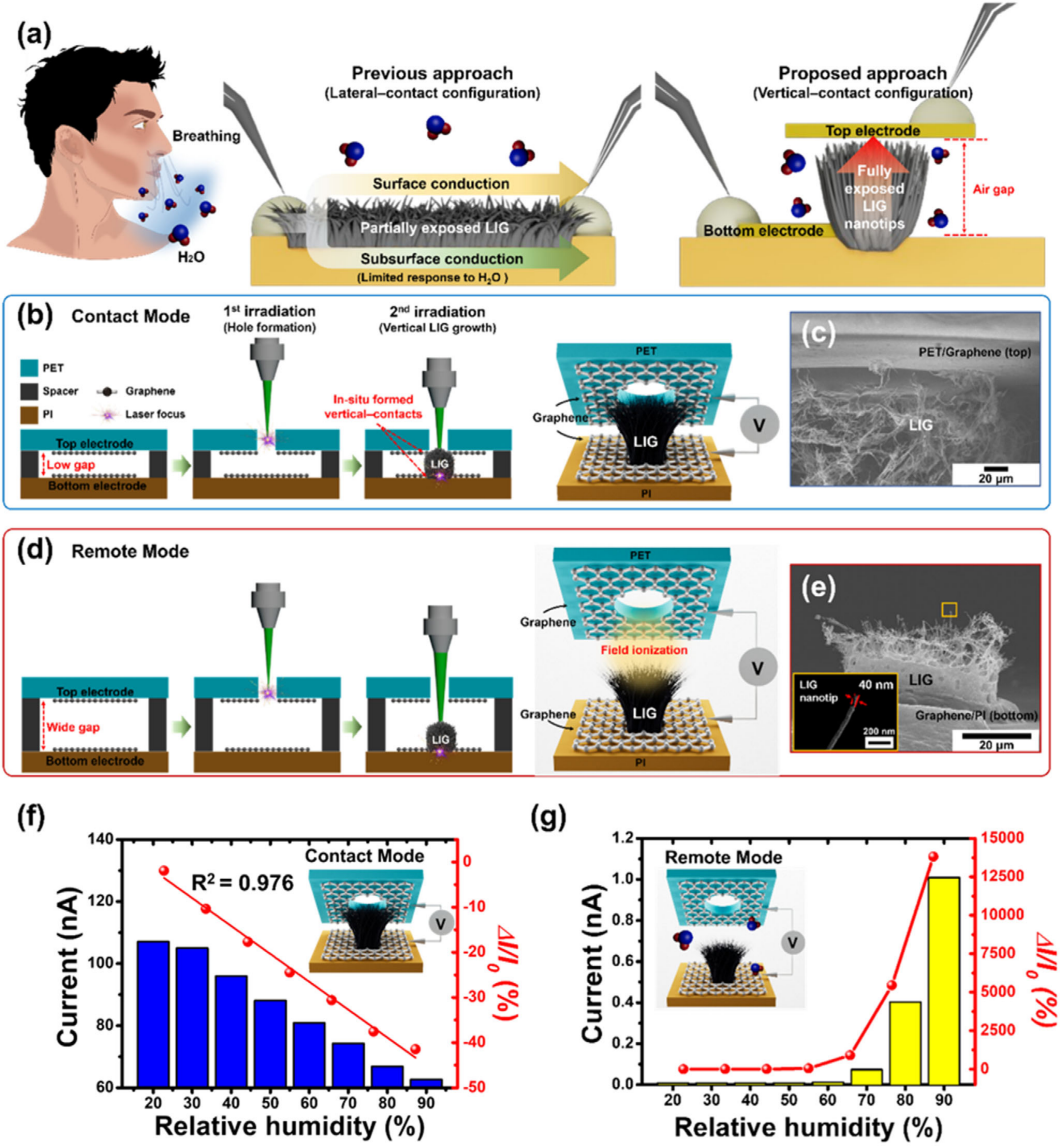
Comprehensive illustration of the operational principles, fabrication processes, and humidity‐sensing performance of LIG‐based sensors in Contact and Remote Modes. a) Schematic comparison between the conventional lateral‐contact and proposed vertical‐contact configurations of LIG‐based humidity sensors. b) Fabrication process for in‐situ‐formed vertical contacts on fully exposed LIG nanotips in Contact Mode. c) Scanning electron microscopy (SEM) image showing the junction between the top graphene electrode and vertically grown LIG nanotips in Contact Mode. d) Fabrication process for the Remote‐Mode sensor based on field ionization. e) SEM image of vertically aligned LIG nanotips operating in Remote Mode. The inset shows a high‐resolution SEM image of an individual LIG nanotip. f) Current response as a function of RH in Contact Mode. Linear fitting of Contact Mode responsivity versus RH data, yielding a high coefficient of determination (R^2^ = 0.9759) g) Current response as a function of RH in Remote Mode.

Conventional LIG‐based humidity sensors typically adopt a lateral‐contact configuration, in which the electrodes are connected to the LIG‐sensing structure to facilitate lateral current flow (**Figure** **1**). These conventional sensors feature a partially exposed structure in which only the surface region interacts with water molecules. A density gradient between the subsurface LIG (embedded in the PI substrate) and the surface LIG limits responsivity,^[^ ^33^ ^]^ as current predominantly flows through the dense subsurface regions rather than the active surface sites. Although vertically grown LIG forms a 3D porous network, its partially exposed structure fundamentally limits the overall responsivity of the sensor.

To address the inherent limitations of conventional lateral‐contact LIG structures, we propose a vertical‐contact configuration that facilitates a fully exposed sensing structure and significantly enhances the humidity‐sensing performance (Figure 1a). In this vertically aligned architecture, the LIG nanotips grown between the top and bottom electrodes are fully exposed to ambient air and surrounded by water molecules, thereby maximizing surface interaction and substantially improving responsivity.

Single‐pulse laser irradiation enables the formation of a vertical‐contact configuration by inducing the localized growth of fully exposed LIG nanotips between the parallel top and bottom graphene electrodes separated by an air gap (Figure 1b,d). This “LIG‐last” process simultaneously forms in‐situ vertical contacts with both electrodes in a self‐aligned manner. Prior to laser irradiation, the CVD‐grown graphene was transferred onto polyethylene terephthalate (PET) (top electrode) and PI (bottom electrode) and then assembled face‐to‐face with a spacer to control the size of the air gap. The fabrication process consists of two steps: 1) a single CO_2_ laser pulse creates a hole (≈150 µm) in the PET/graphene top layer; 2) a second high‐fluence pulse induces the vertical growth of LIG nanotips through the hole, establishing in‐situ‐formed vertical contacts with the top and bottom graphene electrodes. During the first step, PET decomposition produces volatile gases (CO, CO_2_, and CH_4_) and by‐products (terephthalic acid and ethylene glycol).^[^ ^45^, ^46^ ^]^ Unlike PI, PET does not carbonize or form LIG under CO_2_ laser irradiation, preventing unintended LIG conversion and preserving the vertical‐contact architecture. This results in uniform hole formation (≈150 µm diameter) while preserving graphene integrity around the irradiated region, as confirmed by Raman spectroscopy (Figure S1, Supporting Information). Additionally, our previous studies have demonstrated that pre‐transferred graphene on PI surfaces facilitates ohmic contact with the vertically grown LIG structures.^[^ ^47^, ^48^ ^]^

Our LIG‐last process provides a simple, cost‐effective, and precise strategy for forming robust vertical contacts between the vertically aligned LIG nanotips and graphene electrodes. Unlike conventional methods—such as metal deposition with planarization^[^ ^49^ ^]^ or transferring prefabricated electrodes^[^ ^50^ ^]^—which are often complex, time‐consuming, and expensive, our in‐situ approach requires only two single laser pulses to simultaneously induce LIG‐nanotip growth and establish ohmic contacts with both electrodes (Figure S2, Supporting Information). This self‐aligned process can be easily implemented by adjusting the focal length within AutoCAD‐based laser system software, eliminating the need for complex and expensive semiconductor fabrication processes, while maintaining high precision. Additionally, the technique is compatible with roll‐to‐roll processing, allowing for continuous and scalable LIG patterning for large‐area production, further reducing manufacturing costs and enhancing industrial scalability.^[^ ^51^ ^]^

The laser conditions were carefully optimized to enable effective LIG‐nanotip growth, while preventing structural damage during laser scribing. Overlapping laser pulses from raster scanning can cause localized overheating,^[^ ^39^ ^]^ which damages the thin LIG‐nanotip layer, which is essential for maintaining high sensor responsivity. Such thermal degradation weakens the narrow conductive pathways and compromises the structural integrity of the nanotips, thereby reducing their responsiveness to water molecule adsorption. To mitigate this issue, a dot‐patterned, single‐pulse laser irradiation method was implemented by operating the laser in vector mode and adjusting the pulses‐per‐inch (PPI) settings to avoid pulse overlap.^[^ ^52^ ^]^ At fluence levels above 17 J cm^2^, LIG tends to grow vertically, forming fiber‐like structures.^[^ ^39^, ^40^ ^]^ In this study, a laser fluence of 89.9 J cm^2^ was employed to promote the uniform vertical growth of LIG nanotips beyond the PET hole by distributing energy over a wider area of the PI substrate. This approach ensured the formation of a stable conductive interface with the top graphene electrode, which enabled efficient charge transport. Further details on the optimization of the laser fluence parameters are provided in the Supporting Information.

**Figure 2:**
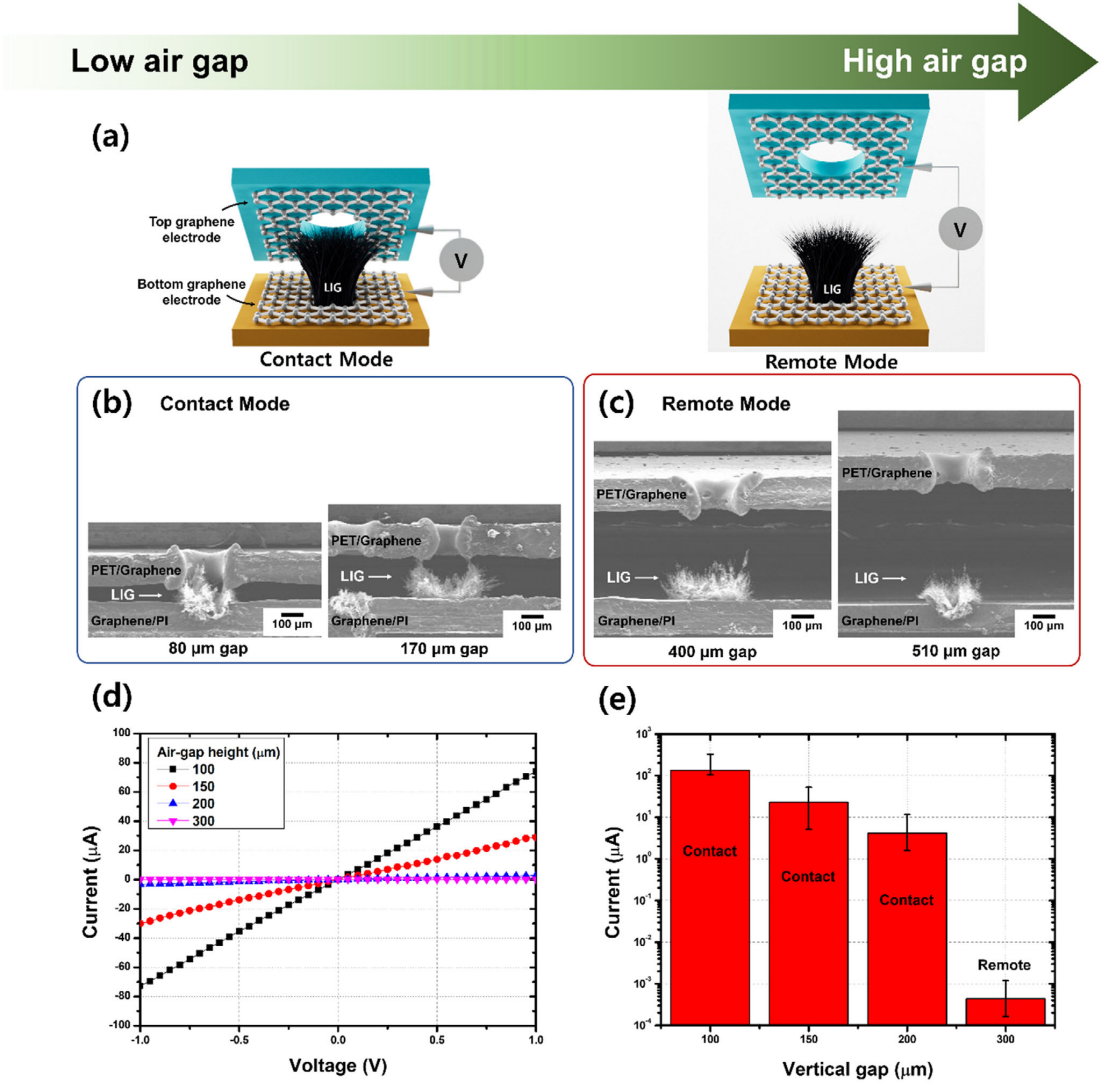
Structural and electrical characterization of vertical LIG‐based humidity sensors as a function of air‐gap height. a) Schematic of sensor operation in the Contact Mode (low air gap) and Remote Mode (high air gap). b) SEM images of LIG nanotips with air gaps of 80 and 170 µm, showing direct physical contact with the top graphene electrode. c) SEM images of the LIG nanotips at air gaps of 400 and 510 µm, demonstrating no physical contact with the top graphene electrode. d) Current–voltage (*I*–*V*) characteristics at different vertical air gaps (100, 150, 200, and 300 µm) measured under a 1 V bias applied to the bottom graphene electrode with the top electrode grounded. e) Bar graph illustrating the relationship between the air‐gap height and current, highlighting the transition from Contact Mode to Remote Mode as the air gap increases. Error bars represent the standard deviation (n = 3).

The sensor's operational mechanism was determined by the air‐gap height between the bottom graphene layer and top graphene electrode, where the LIG nanotips were vertically aligned. Under fixed laser irradiation conditions (89.9 J cm^2^), the LIG nanotips consistently reached a height of ≈250 µm (Figures S3–S5, Supporting Information). The formation of these LIG nanotips is primarily governed by photothermal effects induced by CO_2_ laser irradiation of the PI substrate. As reported in previous studies,^[^ ^39^ ^]^ laser fluences above ≈40 J cm^2^ induce localized melting and vaporization, resulting in vertically aligned fibrous structures instead of planar carbon sheets. This morphological transition—from sheets to fibers to droplets—is driven by the thermal gradient and fluidic dynamics within the laser‐irradiated zone. In our study, although the commercial CO_2_ laser (λ = 10.6 µm) offers limited resolution due to its low duty cycle, we optimized parameters such as fluence (set to 89.9 J cm^2^), scanning speed, and focal position to promote uniform vertical growth. Statistical analysis (Figure S3, Supporting Information) indicated that over 80% of the fabricated nanotips successfully reached the top electrode at an air gap of 230 µm. SEM images (**Figure** **2**; Figure S5, Supporting Information) confirmed that the nanotips, though occasionally bent upon contact, remained structurally intact without damaging the PET substrate. Furthermore, as shown in Figure S4 (Supporting Information), air gaps below 195 µm ensured consistent tip–electrode contact, while gaps exceeding 290 µm resulted in incomplete connection. Therefore, an air gap of 230 µm was selected as an optimal compromise between maximizing nanotip length and ensuring device reliability. Although longer nanotips could theoretically improve sensitivity by extending the conduction path, excessive growth may reduce structural consistency and sensor reproducibility.

When the air gap is minimized (less than the nanotip height), the LIG nanotips make direct contact with the top graphene electrode, forming a vertical conductive network that enables efficient charge transport through direct electrical conduction, referred to as Contact Mode (Figure 1c). In contrast, increasing the air gap beyond the nanotip height prevented physical contact between the nanotips and top electrode. In this case, the high‐aspect‐ratio LIG nanotips terminate below the top graphene electrode without connection, enabling Remote‐Mode operation based on field ionization rather than direct conduction (Figure 1e). By precisely adjusting the air‐gap height, the device can be selectively configured to operate in either Contact Mode or Remote Mode, providing versatile control over the sensor's electrical characteristics and humidity‐sensing performance.

The two operational modes exhibited opposite electrical responses to humidity variations, highlighting their fundamentally distinct sensing mechanisms (Figure 1f,g). In Contact Mode, the thin conductive pathway (on the order of tens to hundreds of nanometers), formed at the LIG‐nanotip region in contact with the top graphene electrode, exhibits high responsivity to moisture adsorption owing to its fully exposed structure. As the RH increases, the water molecule adsorption modulates the resistance of this narrow conductive pathway, resulting in a reduction in the current (Figure 1f). However, in the Remote Mode, the current increased with increasing RH (Figure 1g). In this configuration, the nanotip architecture generates highly localized electric fields at the tip apexes, which enhances the field ionization. As the humidity levels increase, the ionized electrons are further accelerated and collide with water molecules, triggering impact ionization, which results in substantial current amplification. A more detailed explanation of this field‐ionization mechanism is provided in the following section.

These complementary sensing mechanisms achieve an unprecedented level of responsivity, reaching 40% in Contact Mode and an exceptional 14 000% in Remote Mode, across a RH range of 20%–90%, significantly surpassing the performance of conventional LIG‐based humidity sensors (detailed in **Table** **1**). The adjustable air‐gap design enables targeted sensor optimization for specific humidity ranges, thereby demonstrating significant versatility for diverse environmental monitoring applications. Notably, this fabrication method, validated through precise experimental characterization, markedly enhances the humidity‐sensing performance of LIG without requiring additional functionalization or complex post‐processing. Thus, it presents a simple, scalable, and highly effective approach for the development of next‐generation ultra‐sensitive humidity sensors.

**Table 1:** Comparison of reported LIG‐based humidity sensors.

LIG‐based humidity sensor	Type	RH range [%]	Responsivity [%]	Sensitivity [%/%RH]	Response/recovery time [s]	LIG functionalization	Substrate materials
Bulk pattern^[^ ^41^ ^]^	Resistive	10–90	0.104	0.0013	95/637	Pristine	Filter paper (cellulose)
Separated line array^[^ ^62^ ^]^	Resistive	30–90	39	0.65	0.8/7	Pristine	Ethanol‐socked PI
Meander pattern^[^ ^63^ ^]^	Resistive	10–70	0.03 0.48	0.0005 0.008	364/297 180/190	Hydrophilicity enhanced LIG	PI
Meander pattern^[^ ^64^ ^]^	Resistive	40–80	−0.68 ± 0.20	−0.017 ± 0.005	78/65	Pristine	PEI^a)^
IDE pattern ^[^ ^65^ ^]^	Resistive	6–98	166.47	1.81	0.1/0.1	MXene‐silk composite coating	PI
Meander pattern ^[^ ^42^ ^]^	Resistive	40–80	0.6	0.015	4.2/6.8	Pristine	Cork
Contact mode (**This work**)	Resistive	20–90	−40.6	−0.58	1.2/1.2	Pristine	PI
Remote mode (**This work**)	Field ionization	60–90	14000 (*Exponential*)	N/A (*Exponential*)	0.3/0.3	Pristine	PI

^a)^ Polyetherimide.

### 2.2 Effect of Air‐Gap Height on Electrical Characteristics

The air‐gap height plays a critical role in the design of the sensor because it directly influences the electrical contact pathway and determines the operational mechanism between the LIG nanotips and top graphene electrode (**Figure** **2**a). A smaller air gap increased the contact area between the LIG nanotips and top graphene layer (Figure 2b), thereby reducing the resistance and enabling a higher current flow. Furthermore, additional laser irradiation promoted LIG formation, resulting in a linear increase in the current (Figure S6, Supporting Information). These results are consistent with the resistivity law, confirming that the LIG structure functions as an effective current pathway between the two electrodes.^[^ ^53^, ^54^ ^]^ Conversely, as the air gap increased, the physical contact area between the LIG nanotips and top graphene electrode gradually decreased (Figure 2b), leading to a corresponding reduction in the current (Figure 2d). Once the air gap exceeded the height of the LIG nanotips, the conductive pathway was completely severed, resulting in open‐circuit conditions.

SEM imaging confirmed this structural transition and verified that two distinct sensor configurations could be selectively designed depending on the air‐gap height (Figure 2b,c). Under a fixed laser fluence of 89.9 J cm^2^, the LIG nanotips maintained direct contact with the upper graphene electrode at air gaps of 80 and 170 µm (Figure 2b), thereby ensuring a stable electrical connection. However, at larger air gaps of 400 and 510 µm, the LIG nanotips and upper graphene electrode were completely separated (Figure 2c), preventing direct charge transport.

To experimentally validate this relationship, the *I*–*V* characteristics were measured between the two graphene electrodes (Figure 2d,e). The highest current was recorded at an air gap of 100 µm, where the LIG nanotips remained in direct contact with the top graphene electrode, thus enabling efficient charge transport. As the air gap increased to 150 and 200 µm, the current gradually decreased because of the reduced contact area. Once the air gap exceeded the height of the LIG nanotips, physical contact between the two electrodes was lost, resulting in a drastic decrease in the current. For example, at an air gap of 300 µm, under an applied voltage of 1 V and RH below 50%, the detected current decreased to ≈5 pA, indicating a complete open‐circuit condition in which the charge transport was entirely blocked

Compared with conventional LIG‐based humidity sensors, which typically operate with fixed electrode configurations, the proposed tunable air‐gap design can dynamically adjust the air gap, providing an additional degree of optimization. This tunable design enables precise sensor calibration for specific humidity ranges, significantly enhancing adaptability to diverse environmental conditions.

### 2.3 Humidity‐Sensing Performance in Contact Mode

**Figure 3:**
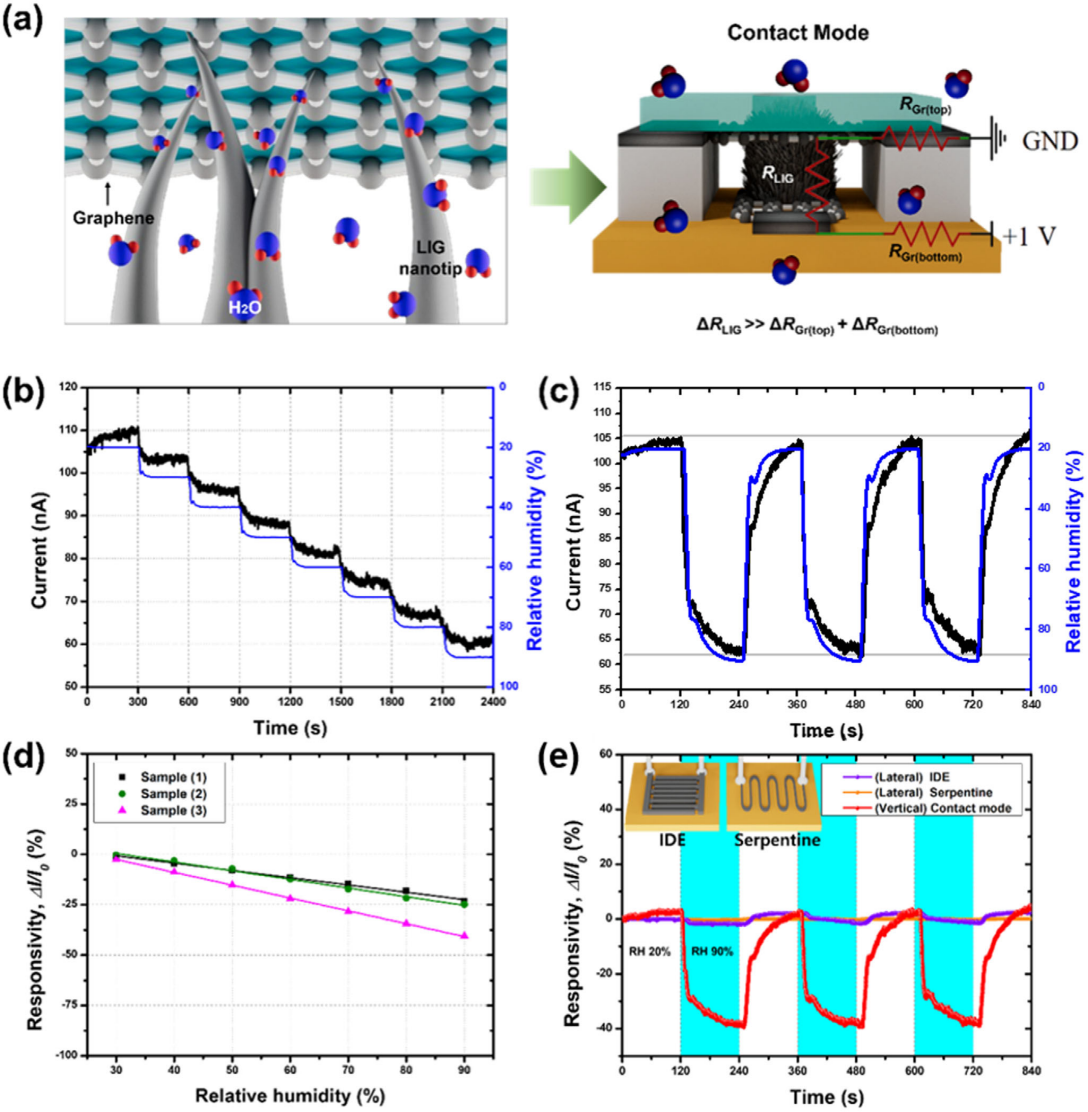
Schematic and performance characterization of the LIG‐based humidity sensor operating in Contact Mode. a) Schematic of humidity‐sensing mechanism of LIG nanotips (left) and equivalent circuit model of fabricated Contact Mode sensor (right). The resistance of LIG nanotips (*R*_LIG_) primarily changes owing to the adsorption of water molecules. During the measurements, a voltage of 1 V was applied to the bottom graphene electrode, and the top graphene electrode was grounded at a controlled temperature of 20 °C. b) Time‐resolved current response of the Contact Mode sensor under varying RH levels. The blue line represents the RH measured using a commercial reference sensor. c) Reproducible current responses of the Contact Mode sensor under cyclic RH variations between 20% and 90%. d) Responsivity profiles of three different Contact Mode sensors fabricated using the same method, showing consistent responses across varying RH levels (−29.5 ± 9.6%). e) Comparative responsivity of conventional lateral‐contact sensors (interdigitated electrode (IDE) and serpentine patterns) and the proposed vertical‐contact sensor (Contact Mode) measured across RH variations from 20% to 90%.

The humidity sensor operating in Contact Mode detects resistance changes in the LIG nanotips, which are fully surrounded by water molecules under varying RH conditions (**Figure** **3**a). The electrical behavior of the LIG‐nanotip‐based sensor operating in Contact Mode was analyzed using a circuit model, as illustrated in Figure 3a (right). The total resistance of the sensor was expressed as the sum of *R*_LIG_, *R*_Gr(top)_, and *R*_Gr(bottom)_, where *R*_LIG_ corresponds to the resistance of the active LIG‐nanotip‐sensing region, and *R*_Gr(top)_ and *R*_Gr(bottom)_ represent the resistances of the top and bottom graphene electrodes, respectively. Because the LIG nanotips exhibited significantly higher responsivity to humidity variations, the change in the total resistance was predominantly influenced by the change in resistance in the LIG‐nanotip region (Δ*R*_LIG_), following the relation Δ*R*_LIG_ >> Δ*R*_Gr(top)_ + Δ*R*_Gr(bottom)_. To verify whether the current change in the sensor originated from the LIG nanotips rather than from the graphene electrodes or the bulk LIG embedded in the PI substrate, the current responses to RH variations (20%–90%) were experimentally analyzed. The results demonstrated that both the graphene electrodes and bulk LIG exhibited minimal current changes (≈1%), indicating their negligible contribution to the overall sensor responsivity (Figures S7 and S8, Supporting Information). These findings support the conclusion that the thin current pathways formed by the LIG nanotips play a dominant role in enhancing sensor responsivity.

The Contact Mode sensor exhibited a highly linear decrease in current with increasing RH (Figure 3b). This decrease in current, attributed to the absorption of water molecules, is consistent with previous results from LIG‐based resistive‐type humidity sensors,^[^ ^55^, ^56^ ^]^ confirming that a similar sensing principle governs their operation. Figure 3c further illustrates the strong correlation between the Contact Mode sensor and commercial reference sensor, confirming its low hysteresis characteristics. As shown in Figure 3d, the three independently fabricated sensors displayed similar electrical behaviors in response to RH variations. Their responsivities, measured over a RH range of 20% to 90%, were −40.8%, −25.3%, and −22.5%. The average responsivity was −29.5% with a standard deviation of 9.6% (Figure S9, Supporting Information). The Contact Mode sensor also maintained consistent responsivity to RH changes over a temperature range of 24–42 °C, further validating its reproducibility and reliability (Figure S10, Supporting Information). Additionally, long‐term stability tests demonstrated that the sensor exhibited no significant performance degradation over 7 days at 90% RH. Moreover, it maintained a consistent response even under abrupt humidity cycling between 20% and 90% at 60‐s intervals for over 5000 s (Figures S11 and S12, Supporting Information), confirming its excellent durability for continuous operation in dynamic environments. The sensor also demonstrated reliable self‐recovery behavior under mechanical pressure, consistently returning to its baseline current after repeated pressure application and release (Figure S13, Supporting Information), thereby highlighting its mechanical durability.

To evaluate the responsivity improvements achieved by our vertical‐contact configuration with fully exposed LIG nanotips, we conducted a comparative analysis with conventional approaches that employ a lateral‐contact configuration. Two types of conventional LIG‐based humidity sensors with lateral‐contact configurations were fabricated: one with an IDE pattern and the other with a serpentine pattern (insets in Figure 3e). The IDE design comprises 10 finger electrodes, each 2‐mm wide with a 0.5‐mm inter‐electrode spacing (Figure S14, Supporting Information). This structure facilitates electrically conductive pathways between the LIG fibers grown on the electrodes, thereby enabling efficient charge transport. Alternatively, the serpentine design features 1‐mm‐wide electrodes spaced 1.5‐mm apart, incorporating six periodic bends (Figure S15, Supporting Information). This configuration extends the effective conduction path and increases the active surface area, thereby enhancing responsivity. Although both the IDE‐ and serpentine‐patterned sensors possessed relatively large surface areas, they exhibited only minor current changes of 0.15% (from 189 to 188.709 µA) and 0.35% (from 47.4 to 47.236 µA), respectively, over the same RH range (Figure 3e). In contrast, our LIG‐nanotip‐based Contact Mode sensor achieved a substantial 40% change in current (from 0.102 to 0.063 µA) under identical conditions. This corresponds to 266‐ and 114‐fold responsivity enhancements compared to those of the IDE and serpentine‐patterned sensors, respectively, highlighting its superior performance in precise humidity detection. Furthermore, the vertical LIG sensor exhibited excellent linearity with a correlation coefficient of R^2^ = 0.976 (Figure S16, Supporting Information), supporting its reliability for accurate and consistent humidity measurements. A more detailed discussion of the high responsivity of the vertical LIG humidity sensor is provided in the following section.

### 2.4 Mechanistic Insights into Enhanced Humidity Sensing via Fully Exposed Ultrathin Current Pathways in Contact Mode

Humidity sensors utilizing LIG typically operate through the adsorption of water molecules onto the LIG surface, where dipole interactions induce charge redistribution or facilitate electron exchange, leading to measurable variations in resistance. Additionally, hydrophilic functional groups, such as –COOH and –OH, further enhance water molecule adsorption, thereby improving sensor responsivity.^[^ ^55^, ^57^ ^]^ These adsorbed water molecules perturb the electronic properties of the sp^2^‐hybridized carbon network by altering the charge distribution or introducing doped charges, thereby modifying the conductivity of the LIG material.

However, conventional LIG sensors often suffer from reduced responsivity owing to repeated laser irradiation, which results in multilayered stacking and excessive interconnectivity. This fabrication process leads to dominant subsurface current pathways with limited interaction at the surface, thereby substantially diminishing resistance changes in response to humidity variations. For example, common lateral‐contact designs—such as IDE and serpentine patterns—exhibit high baseline conductivities (IDE ≈ 47 µA; serpentine ≈ 189 µA), which constrain their responsiveness to water molecule adsorption.

In contrast, the high responsivity of the fabricated Contact Mode sensor is attributed to the fully exposed ultrathin current pathways formed by the vertically aligned LIG nanotips. This structure is achieved through a single‐pulse laser process that leverages the Gaussian energy distribution, resulting in tapered and porous LIG‐nanotip arrays with fully exposed conduction pathways. The strongest carbonization occurs at the center of the laser spot, with progressively decreasing thermal energy toward the edges, creating a gradually tapered structure (Figure 3a, left). Furthermore, the upper regions of the LIG nanotips experience greater heat dissipation and gasification than the substrate‐proximal regions, leading to the formation of a highly porous 3D structure.^[^ ^52^ ^]^ This morphology maximizes the adsorption of water molecules and facilitates efficient charge‐carrier modulation, resulting in a significantly enhanced responsivity. These results are consistent with previous reports on microfiber‐based gas sensors, which demonstrated that reducing conduction pathways in nanostructured materials improves sensing performance.^[^ ^58^ ^]^ This structural morphology enhances physical interaction with water molecules and directly influences the underlying conduction mechanism of the sensor. In the proposed device, conduction is primarily electronic, occurring within the sp^2^‐hybridized carbon network of LIG, which results in linear *I–V* characteristics and rapid response and recovery times. This behavior differs from slower, nonlinear responses observed in ion‐ or proton‐mediated conduction, confirming that our sensor operates via surface‐dominated electronic transport.^[^ ^59^, ^60^, ^61^ ^]^

The unique combination of a fully exposed active surface provided by ultrathin nanotip structures and an in‐situ‐formed vertical‐contact configuration highlights the superior humidity‐sensing capability of the Contact Mode sensor.

### 2.5 Humidity‐Sensing Performance in Remote Mode

**Figure 4:**
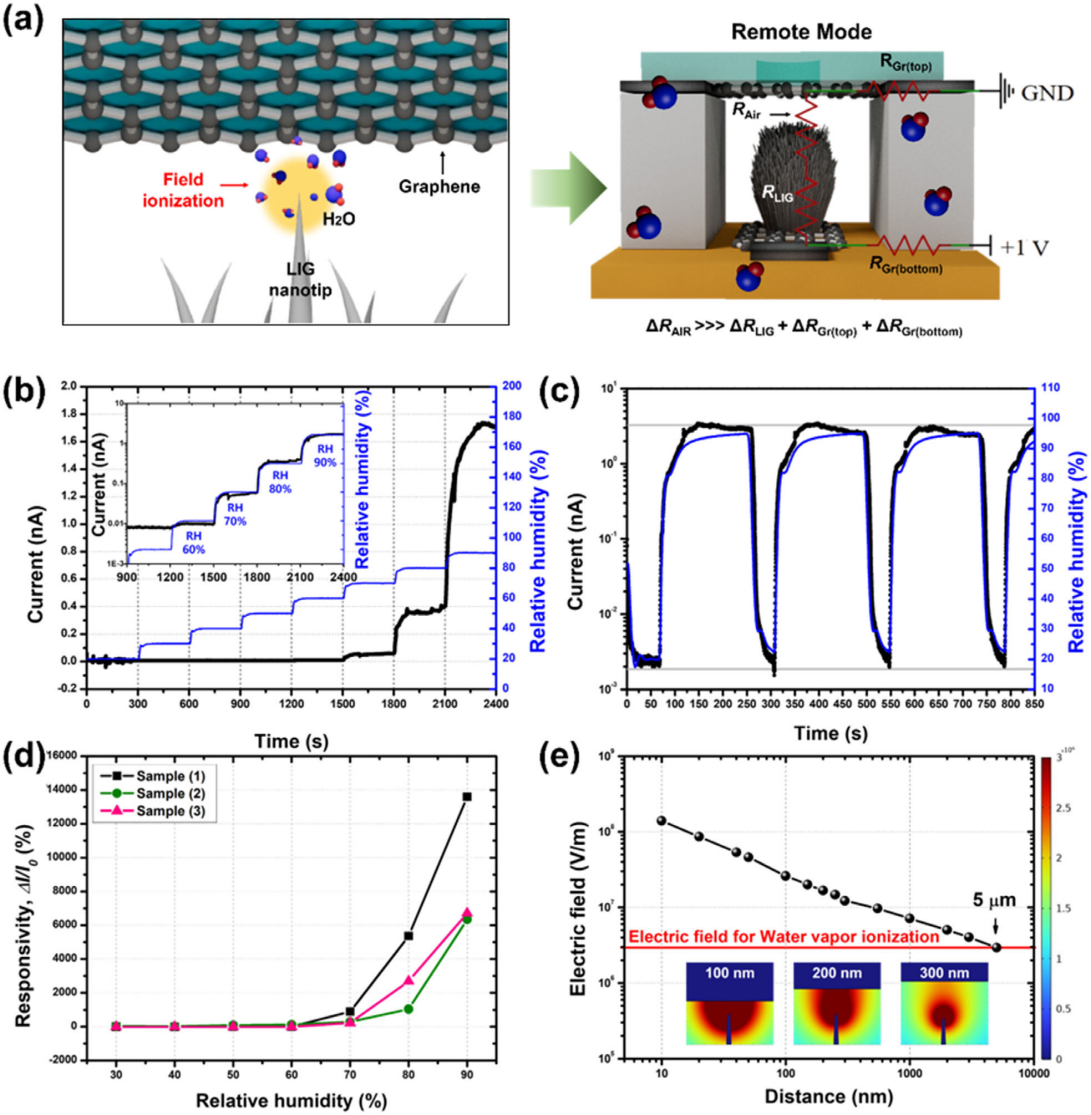
Schematic and performance characterization of the LIG‐based humidity sensor operating in Remote Mode. a) Schematic of the humidity‐sensing mechanism in the Remote‐Mode configuration, where the LIG nanotips and upper graphene electrode are spatially separated (left), and the corresponding equivalent circuit model (right). b) Current response of the Remote‐Mode sensor under varying RH levels. The blue line indicates the RH measured using a commercial reference sensor. The inset shows the current response plotted on a logarithmic scale for the 50%–90% RH range. c) Reproducible current responses of the Remote‐Mode sensor to cyclic RH variations between 20% and 90%. d) Responsivity characteristics of three independently fabricated Remote Mode sensors under varying RH levels, demonstrating consistent performance (8886 ± 4084%). e) Simulated electric‐field distribution as a function of the separation distance (*d*) between the top graphene electrode and a single LIG nanotip, calculated using COMSOL finite element analysis.

The LIG‐nanotip‐based Remote‐Mode humidity sensor operates through a fundamentally different mechanism than the Contact Mode sensor, with exceptionally high responsivity and exponentially increasing current (R^2^ = 0.998, Figure S16, Supporting Information) response within a specific RH range. As illustrated in **Figure** **4**a (right), the circuit model of the Remote‐Mode sensor includes *R*_Air_, which represents the air resistance between the top graphene electrode and LIG nanotips. Under dry conditions, *R*_Air_ remained extremely high, effectively preventing current flow. However, at elevated humidity levels (>60% RH), ionization occurs because of the strong localized electric field at the LIG nanotips, which allows current to flow through *R*_Air_. Because the mode is governed by the ON/OFF switching behavior of *R*_Air_, the overall resistance change follows the relation: Δ*R*_Air_ ≫ Δ*R*_Gr(top)_ + Δ*R*_Gr(bottom)_ + Δ*R*_LIG_.

As shown in Figure 4b, the current remained negligible until the RH reached ≈50%, then increased exponentially above 60% RH. On a logarithmic scale, the current exhibited a linear relationship with the RH (Figure 4b). Considering that the detection limit of our measurement setup was ≈1.5 pA, it is likely that the currents generated below 60% RH were below this threshold. Using high‐resolution instrumentation capable of detecting sub‐picoampere currents would extend the effective detection range of the Remote‐Mode sensor to lower humidity levels, enabling a more precise evaluation of its detection limit (Figure S17, Supporting Information). Furthermore, the experimental results demonstrate that the Remote‐Mode sensor maintains excellent agreement with a reference humidity sensor, confirming its low hysteresis (Figure 4c).

The Remote‐Mode sensor also exhibited stable humidity detection across a wide temperature range (15–25 °C), maintaining consistent responsivity despite temperature variations and effectively isolating humidity changes from temperature‐induced effects (Figures S18 and S19, Supporting Information). In addition, the sensor maintained consistent performance without degradation over a 7‐day period at 90% RH and 20 °C (Figure S20, Supporting Information). Furthermore, it exhibited stable current responses with low standard deviations during repeated humidity variations from 20% to 90% at 1 min intervals for 5000 s, thereby confirming the robustness of the device (Figure S21, Supporting Information).

Furthermore, the Remote‐Mode sensor exhibited exceptional gas selectivity. It effectively distinguished water molecules (or H_2_O) from interfering gases, such as NO_2_ (100 ppm) and NH_3_ (50 ppm), owing to the low ionization energy, high polarity, and high dielectric constant of water molecules. Specifically, the Remote Mode demonstrated selectivity enhancements of 31‐fold over NO₂ and 2580‐fold over NH_3_ (Figure S22, Supporting Information). In comparison, conventional LIG‐based humidity sensors exhibit significantly lower selectivity. The IDE‐patterned sensor showed only 1.76‐ and 18‐fold greater detection performance for water molecules than for NO_2_ and NH_3_, respectively. In contrast, the serpentine‐patterned sensor exhibited 0.17‐ and 8.5‐fold lower selectivity for water molecules relative to NO_2_ and NH_3_, respectively.

As shown in Figure 4d, the three independently fabricated Remote Mode sensors exhibited consistent electrical responses to changes in RH, with the current exponentially increasing. The Remote Mode achieved an exceptional responsivity of ≈14 000%, with the current increasing from 0.012 to 1.735 nA over a RH range of 20%–90%. The responsivities of the three sensors were 13 598%, 6711%, and 6350% (Figure S23, Supporting Information), with an average responsivity of 8886%, demonstrating excellent reproducibility. This performance surpasses all previously reported LIG‐based humidity sensors (Table 1). This exceptional responsivity is attributed to the pre‐breakdown current induced by field and subsequent impact ionization, both initiated by the strong electric field concentrated at the sharp nanotip structures of the vertically grown LIG structures. Under high‐humidity conditions, the intense localized electric field at these nanotips ionizes the surrounding water molecules, thereby generating free charge carriers. These carriers undergo impact ionization, further amplifying charge density and enabling a significant current increase through *R*_Air_. This electric‐field enhancement phenomenon was further analyzed through theoretical studies and electric‐field simulations, as detailed in the following section.

### 2.6 Mechanistic Insights into Field Ionization‐Driven Exponential Current Increase in Remote Mode

The LIG‐nanotip structures fabricated using a single laser pulse (fluence: 89.9 J cm^2^) exhibited sharp nanometer‐scale tip diameters, which significantly enhanced the localized electric fields. As the radius of curvature decreased, the surface charge density increased, thereby amplifying the local electric‐field strength. This phenomenon is widely observed in high‐aspect‐ratio materials such as carbon nanotubes,^[^ ^66^ ^]^ gold nanowires,^[^ ^67^ ^]^ and manganese nanorods,^[^ ^68^ ^]^ and is intrinsic to LIG due to its unique laser‐induced nanostructure.^[^ ^69^ ^]^ These nanotips generate intense localized electric fields exceeding the ionization threshold of water molecules (3 × 10^6^ to 1 × 10^7^ V/m).^[^ ^70^, ^71^, ^72^ ^]^

Upon voltage application, these intense electric fields induce field ionization, whereby H_2_O molecules are ionized into H_2_O^+^ ions and free electrons—via direct ionization or quantum tunneling—without direct electrode‐to‐molecule contact, as described in Equation (1):

(1) $$H_{2}O\to H_{2}O^{+}+e^{-}$$

The free electrons generated through field ionization were accelerated by the applied electric field and collided with additional H_2_O molecules, triggering impact ionization and generating more free electrons. This cascading process, known as the Townsend discharge, exponentially amplifies the current. According to the humidity‐dependent current model, the pre‐breakdown current *I* in a Townsend discharge across an electrode gap distance *d* is expressed by Equation (2):^[^ ^73^, ^74^ ^]^

(2) $$I=I_{0}e^{\alpha d}=I_{0}{e^{\alpha }}^{\left[H2O\right]\cdot d}$$

Here, *I*₀ represents the initial current generated at the LIG nanotips, and *α* denotes the Townsend ionization coefficient, which is related to the collision frequency and electron mean free path. The sensor current was exponentially amplified as the water molecule concentration ([H₂O]) increased with RH.

Precise control of the electrode gap (*d*) is critical. Although the theoretical model suggests that increasing *d* increases the collision frequency, in practice, the electric‐field strength decreases with larger gaps, thereby reducing the ionization efficiency. Conversely, reducing the gap enhances the local electric field and significantly improves the ionization efficiency. Further details of the collision ionization efficiency are provided in the Supporting Information. Finite‐element method simulations confirmed that effective ionization occurred only when the electrode gap was below 5 µm at an applied voltage of 1 V (Figure 4e, Table S1 and Figures S24 and S25, Supporting Information).

Experimental validation at 1 V (below the breakdown voltage threshold), as shown in Figure 4b, confirmed exponential current amplification with increasing RH, supporting the proposed impact ionization mechanism.^[^ ^70^, ^71^ ^]^ Stability tests conducted under applied voltages ranging from 1 to 100 V at a constant RH revealed electrical breakdown only under extreme conditions (90% RH, voltage exceeding 59 V; Figure S26, Supporting Information). This was consistently observed in other sensors fabricated using the same process, demonstrating stable device operation even under high‐voltage conditions.

### 2.7 Respiratory‐State Monitoring

**Figure 5:**
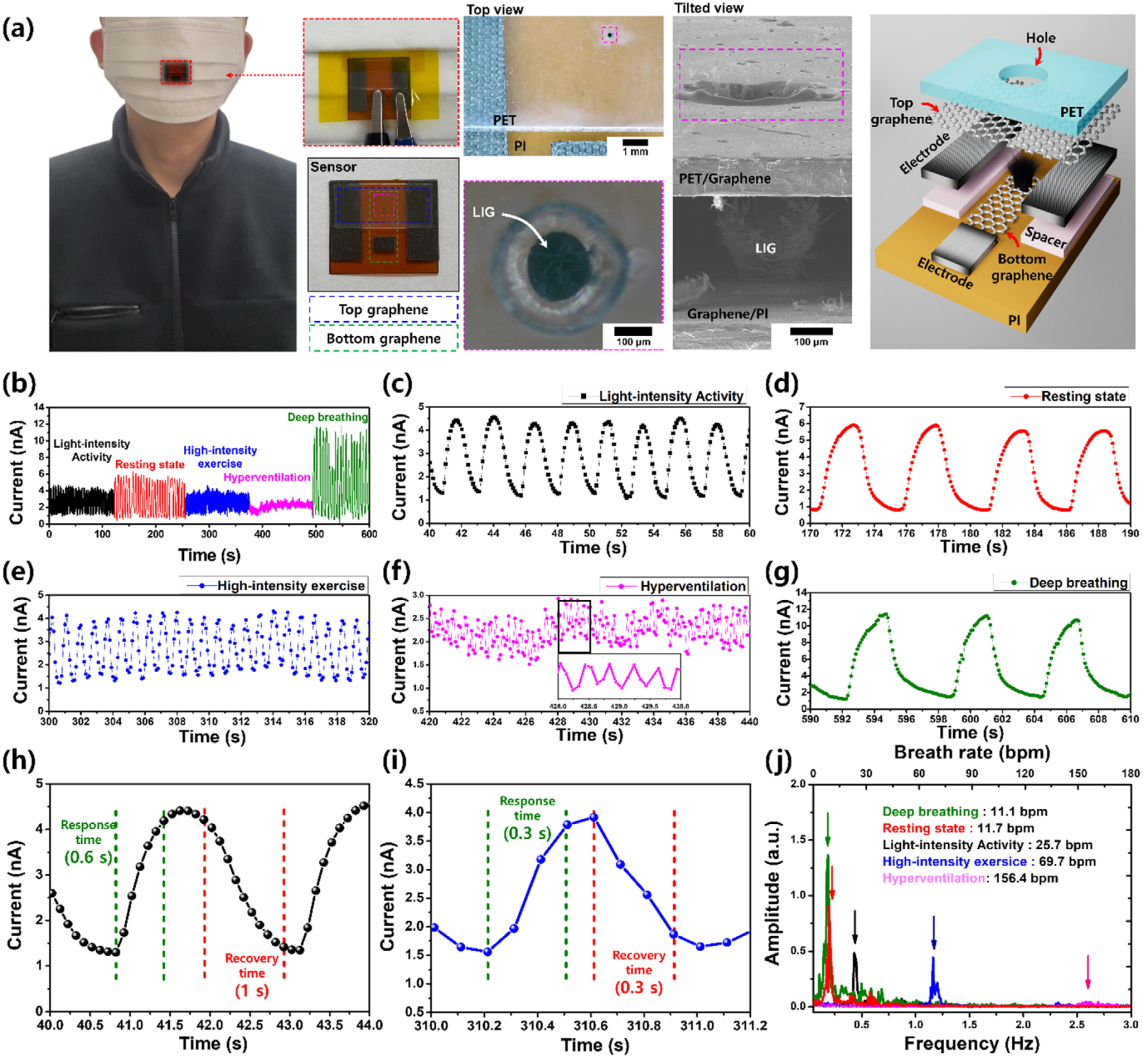
Design, integration, and real‐time monitoring performance of the LIG‐based humidity sensor embedded in a face mask. a) Photograph, optical microscopy image, SEM image, and schematic of the humidity sensor integrated into a wearable face mask worn by a volunteer. b) Real‐time sensor responses under various breathing conditions. Sensor response signals during c) light‐intensity exercise breathing, d) resting‐state breathing, e) high‐intensity exercise breathing, f) hyperventilation, and g) deep breathing. h) Response and recovery times for inhalation and exhalation during light‐intensity exercise. i) Response and recovery times for inhalation and exhalation during high‐intensity exercise. j) Fast Fourier Transform‐based analysis of breathing amplitudes and frequencies under different respiratory conditions.

To evaluate the real‐world applicability of the LIG‐nanotip‐based humidity sensors, both Contact‐ and Remote‐Mode sensors were individually integrated into wearable face masks for real‐time respiratory monitoring (**Figure** **5**a). Each sensor was tested separately to assess its ability to detect humidity variations across different respiratory conditions. Ambient air primarily enters the sensing region through the lateral gaps between spacers rather than the laser‐drilled hole in the PET film. This is attributed to the significantly larger cross‐sectional area of the side openings (≈1.15 mm^2^) compared to the laser hole (≈7.85 × 10^−^^3^ mm^2^), making the sides the main pathway for air exchange under both chamber‐based and respiration‐monitoring conditions.

The performance of both Contact Mode and Remote Mode sensors is significantly influenced by the contact area and distance between the vertically grown LIG nanotips and the graphene layer. Therefore, a 125‐µm‐thick substrate was employed in the final design to prevent unintended signal instability caused by variations in contact area or changes in the interelectrode distance (*d*) due to substrate bending or breathing‐induced pressure.

The Contact Mode sensor successfully detected normal breathing patterns, recording a stable respiratory rate of 15.9 breaths per minute (bpm), within the average adult range of 12–20 bpm, with a response and recovery time of 1.2 s each (Figure S27, Supporting Information).

Notably, the field ionization‐based Remote‐Mode sensor demonstrated exceptionally fast response and recovery times, enabling accurate detection across various respiratory conditions—including resting, deep breathing, exercise, and hyperventilation (Figure 5b–g). During low‐ and high‐intensity exercises, the response times were ≈0.6 and 0.3 s, respectively, with corresponding recovery times of 1 and 0.3 s (Figure 5h,i).

Furthermore, the Remote‐Mode sensor maintained stable performance even at ultra‐fast breathing rates of up to 156.4 bpm, without exhibiting signal saturation or response lag. Typically, higher breathing rates shorten the inhalation and exhalation cycles, thereby reducing the humidity fluctuations per breath. Although conventional humidity‐based sensors often struggle to detect these minimal variations, the proposed Remote‐Mode sensor overcomes this limitation through its ultra‐high responsivity (14 000%) and rapid response/recovery times of less than 1 s. Consequently, it accurately captured the short and rapid respiratory cycles with high precision.

Additionally, amplitude and frequency analyses using FFT enabled the precise characterization of both breathing intensity and respiratory rate. As shown in Figure 5j, this analysis facilitated the identification of abnormal respiratory patterns—such as apnea or irregular breathing cycles—providing a more comprehensive assessment of respiratory health. These findings demonstrate the practical feasibility and versatility of the proposed humidity sensors in wearable respiratory monitoring applications, offering real‐time, accurate, and noninvasive assessments of respiratory states under various physiological conditions.

## 3 Conclusion 

In this study, we developed highly sensitive humidity sensors for respiratory monitoring, leveraging a novel vertical‐contact architecture featuring in‐situ fabricated, vertically aligned LIG nanotips. Unlike conventional lateral‐contact LIG humidity sensors—such as those employing IDE or serpentine patterns—which suffer from excessive subsurface current pathways and limited surface exposure of active LIG regions, our vertically grown nanotip structures feature fully exposed ultrathin current pathways, significantly enhancing interactions with water molecules. Consequently, the Contact Mode sensor achieved 40% responsivity across a RH range of 20%–90%, representing more than a 100‐fold improvement over conventional designs. Additionally, we introduced a Remote‐Mode sensing mechanism that leverages the sharp curvature of the LIG nanotips to induce field ionization and subsequent impact ionization at voltages as low as 1 V. This approach yielded an exceptional responsivity of ≈14 000%, which is among the highest values reported for LIG‐based humidity sensors to date. The proposed LIG‐last process further simplifies sensor fabrication by employing only two single laser pulses, eliminating the need for complex, time‐consuming, and expensive semiconductor techniques. The fabricated sensors exhibited excellent temperature stability, gas selectivity, and long‐term durability, highlighting their potential for diverse applications. Finally, we demonstrated the practical utility of the sensors by integrating them into wearable face masks for real‐time monitoring of the respiratory state. The sensors exhibited rapid response and recovery times (<1 s), reliable performance under dynamic conditions, such as hyperventilation and high‐frequency breathing (up to 156 bpm), and long‐term operational stability. Although the proposed approach achieved outstanding performance, precisely controlling the vertical growth height of the LIG nanotips remains challenging, potentially leading to variability in device responsivity. Future studies should focus on minimizing growth variations by optimizing the laser parameters and process conditions to enhance reproducibility and uniformity. By introducing a cost‐effective fabrication method and uncovering novel sensing mechanisms, this study underscores the potential of vertical‐contact LIG‐nanotip sensors as next‐generation platforms for wearable healthcare devices, environmental monitoring, and industrial applications, offering a highly sensitive, scalable, and real‐time solution for detecting humidity.

## 4 Experiment Section

### Graphene‐Transfer Process onto PI and PET Films

Monolayer graphene grown by CVD on Cu foil (thickness: 18 µm) was sourced from Chamgraphene Co., Ltd. (Suwon, South Korea). A PI film (Kapton HN, thickness: 125 µm) was procured from DuPont (Wilmington, Delaware, USA), and a PET film (thickness: 125 µm) was procured from Film Bank (Siheung, South Korea). To transfer CVD‐grown monolayer graphene onto the PI or PET film, the graphene‐coated Cu foil was first laminated onto a thermal release tape (TRT).^[^ ^75^ ^]^ The Cu layer was then etched using a 0.1 M ammonium persulfate solution until it was completely removed. After etching, the residual contaminants were removed by rinsing with deionized water, followed by drying with N_2_. The resulting graphene/TRT film was then laminated onto either the PI or PET film using a laminator. The TRT film was removed by heating the assembled structure on a hot plate at 100 °C for 1 min, yielding graphene/PI and graphene/PET films. A schematic of the graphene‐transfer process is shown in Figure S28 (Supporting Information).

### Fabrication of LIG‐Based Humidity Sensors

Graphene/PI (bottom electrode) and graphene/PET (top electrode) structures were prepared using the TRT‐assisted graphene‐transfer method. A 20 mm × 20 mm PI film was used as the substrate, onto which a 5 mm × 15 mm graphene layer was transferred at its center. For the top electrode, a 7 mm × 10 mm PET film was prepared with graphene transferred over its entire surface. Next, to assemble the sensor and control the air‐gap height, a thick double‐sided PI tape (5 mm × 15 mm, thickness: 50 µm) was laminated onto both ends of the PI substrate. The number of laminated layers was adjusted to control the air‐gap height and prevent direct contact between the electrodes. This configuration ensured electrical isolation, preventing unintended current pathways caused by water accumulation on the PI film under high‐humidity conditions. Subsequently, carbon tape (5 mm × 10 mm, thickness: 100 µm) was laminated onto the PI tape to facilitate electrical contact, and the pre‐prepared PET/graphene stack was precisely aligned and attached to ensure reliable electrical contact with the carbon tape. To form the LIG nanotips, a CO_2_ laser system (Universal Laser Systems ULS 2.30; maximum output: 30 W; vector mode scan rate: 10 in/s; wavelength: 10.6 µm) was employed (laser system specifications were shown in Figure S29, Supporting Information). The CO_2_ laser was operated in vector mode using the following parameters: power = 55%, scan rate = 100%, PPI = 130, and focus offset = 0 mm. The first laser pulse was applied to the center of the PET/graphene (top electrode) stack to create a hole. A second laser pulse was adjusted to the focus offset of the graphene/PI (bottom electrode) stack to induce LIG‐nanotip formation, thereby completing the sensor fabrication. The air‐gap height was measured using a digital micrometer caliper (Mitutoyo, coolant‐proof type, IP65 rated; resolution: 0.001 mm) and confirmed via optical microscopy (OM System with SCMOS05100KPB, TYZK, China). A schematic of the 3D sensor configuration was provided in Figure S30 (Supporting Information).

### Characterization

The surface morphologies of the fabricated structures were characterized using field‐emission SEM (Hitachi 4800–1, Hitachi, Japan). Raman spectroscopy (Horiba LabRAM HR‐800, Horiba, Japan) was employed to analyze the structural and compositional properties of the graphene and LIG structures.

### Experimental Setup for Humidity‐Sensing Tests

The fabricated LIG‐based humidity sensors were evaluated in a controlled humidity chamber (MPS‐PTH, NEXTRON, Busan, South Korea). Sensor responses were recorded using a source meter (Keithley 2612B, Keithley Instruments, USA) under a constant bias voltage of 1 V. RH levels were regulated using a humidity control system (HCS‐2 M, NEXTRON, Busan, South Korea) and compared with a reference humidity sensor (HCS‐2 M, NEXTRON, Busan, South Korea) under identical conditions. All measurements were conducted at ambient pressure (1 atm) and a controlled temperature of 20 ± 0.2 °C.

### Volunteer‐Based Evaluation of Respiratory States

To evaluate the real‐time respiratory monitoring capability of the proposed system, a healthy male volunteer (weight = 72 kg; height = 177 cm) performed a series of controlled breathing exercises while wearing a face mask embedded with the sensor. The sensor was integrated into the inner surface of a mask (KleenGuard Soft Clean Mask (K), Yuhan‐Kimberly, Seoul, South Korea) and positioned near the nose to accurately detect humidity changes during respiration. The volunteers met the following inclusion criteria: adults over 20 years of age, non‐smokers, no history of chronic respiratory or cardiovascular diseases or any conditions that may affect exercise performance, and the physical ability to perform the required respiratory tasks. Written informed consent was obtained after a full explanation of the study objectives and procedures was provided. The electrical signals were measured using a source meter (Keithley 2612B, Keithley Instruments, USA) and transmitted to a Windows 10‐based PC (Microsoft, USA) via a GPIB interface. Real‐time data acquisition was performed using LabVIEW software (LabVIEW, National Instruments, USA). The breathing conditions evaluated in the experiment were as follows: light‐intensity exercise (jumping jacks performed at ≈24 counts per minute—defined by one full arm raise per count—for 1 min, followed by 1 min of measurement, totaling 2 min), resting state (measured immediately after exercise in a relaxed, seated posture), high‐intensity exercise (jumping jacks at a maximum sustainable pace of ≈88 counts per minute), hyperventilation (induced by deliberately increasing breathing frequency to simulate rapid, shallow breathing), and deep breathing (inhaling as deeply as possible through the nose and exhaling slowly to simulate slow, full respiratory cycles).

### Environmental Stability Evaluation of LIG Nanotip Structures

Environmental scanning electron microscopy observations confirmed that the LIG nanotip structure remained morphologically stable under various environmental conditions. Specifically, no structural deformation or collapse was observed as the temperature increased from 20 to 50 °C. Moreover, the LIG structure remained unchanged under gradually increasing RH from 20% to 100% (Figure S31, Supporting Information).

### Study Participation

This study involved noninvasive measurements of respiratory humidity using a wearable sensor. Written informed consent was obtained from all participants prior to their participation following a comprehensive explanation of the study's objectives and procedures.

### Statistical Analysis

All statistical analyses were performed to evaluate the reproducibility and consistency of the sensor's performance. No data transformation or outlier removal was applied, and all raw measurement data were analyzed in their original forms. The number of samples used in the statistical analysis was indicated in both the main text and figure legends. Where data from multiple samples were averaged, the results were presented as mean ± standard deviation. All statistical analyses were conducted using Microsoft Excel 365 and OriginPro 8.1.

## Conflict of Interest

The authors declare no conflict of interest.

## Supporting information



Supporting Information

## Data Availability

The data that support the findings of this study are available from the corresponding author upon reasonable request.
