# Supplementary material for: In‐Situ Vertical‐Contact Engineering of Laser‐Induced Graphene Nanotips for Ultra‐Sensitive Humidity Sensors
Source: Small. 2025 Jul 4;21(34):2505017. doi: 10.1002/smll.202505017 (PMC12393033; doi:10.1002/smll.202505017)
Supplement: Supplementary file 1 — Supporting Information [file SMLL-21-2505017-s001.docx]

Supporting Information

**In-Situ Vertical-Contact Engineering of Laser-Induced Graphene Nanotips for Ultra-Sensitive Humidity Sensors**

*Ki Wan Kim, Won Gyun Park, Do-Yeon Lee, Ga-Won Lee, Binghao Wang, and Jae-Hyuk Ahn**

K. W. Kim, W. G. Park, D. Y. Lee, G. W. Lee, and J. H. Ahn

Department of Electronics Engineering, Chungnam National University,

Daejeon 34134, Republic of Korea
E-mail: [jaehyuk@cnu.ac.kr](mailto:jaehyuk@cnu.ac.kr) (Corresponding Author: J. H. Ahn)

B. Wang
School of Electronic Science and Engineering, Southeast University,

Nanjing, Jiangsu 211189, China

**Fluence Under Single-Pulse Irradiation**

Fluence is defined as the amount of energy delivered per unit area. In laser systems, it represents the energy density of the laser beam, indicating the total energy delivered to a unit area. This can be mathematically expressed as follows:

$F=\frac{E_{F}}{A_{F}}$, (S1)

where $F$ denotes the fluence (J/cm²) and *E*_F_ represents the energy delivered by a single laser pulse (J). *A*_F_ indicates the irradiated area (cm²), which is typically determined by the laser’s focusing conditions and has a strong influence on the local energy density.

In laser systems, the energy per pulse *E*_F_ is related to the average power $P$ (W = J/s) and the pulse repetition rate $f$ (Hz) as

$E_{F}=\frac{P}{f}$. (S2)

**Fluence Under Multi-Pulse Overlapping Conditions**

In this study, which utilizes single-pulse irradiation, only one laser pulse is delivered to each position on the sample. This is achieved by setting a low pulse per inch (PPI), which prevents pulse overlap and ensures that each region is irradiated only once. In contrast, conventional lateral-contact structures are typically patterned under continuous multi-pulse irradiation, where laser pulses repeatedly overlap. In such cases, the effective fluence is influenced not only by the pulse energy, but also by the following parameters:

- Scan rate (mm/s): Speed at which the laser beam moves across the surface.
- Pulse repetition rate $f$ (Hz): Frequency at which pulses are fired.
- Effective area *A*_F_ (cm²): Cross-sectional area covered by the scanning laser beam, typically estimated as the product of the beam diameter and scan pitch.

Under multi-pulse overlapping mode, multiple pulses contribute to the fluence delivered along the scanning path. The number of pulses per unit length is given by

$PPI=\frac{f}{Scan Rate}$. (S3)

Therefore, under multi-pulse overlapping conditions, the accumulated energy per unit area (fluence) is expressed as follows (incorporating PPI to account for the number of laser pulses delivered to the same location)—unlike in single-pulse irradiation.

$F=\frac{E_{F}\cdot PPI}{A_{F}}=\frac{P\cdot PPI}{f\cdot A_{F}}$ (S4)

Substituting PPI ​, we obtain

$F\approx\frac{P}{Scan Rate\times A_{F}}$. (S5)

**Responsivity (%)**

Responsivity is defined as the relative change in the current due to a change in relative humidity (RH) and is normalized to the baseline current. It quantifies the sensor’s ability to respond to humidity stimuli.

$Responsivity \left( \% \right)=\frac{I_{RH}-I_{0}}{I_{0}} \times100$ (S6)

*I* _RH_: Current at a specific RH level.

*I* _0:_ Current at baseline (unless otherwise stated, RH = 20%).

**Sensitivity (%/%RH)**

Sensitivity represents the rate of current change (or resistance) per unit change in RH, reflecting how sharply the sensor output responds to changes in RH.

$Sensitivity \left( \%/\%RH \right)=\frac{Responsivity}{\Delta RH}$ (S7)

Δ*RH*: Difference in RH.

**Responsivity** refers to the relative change in current with respect to the baseline current (I₀) following a change in RH, whereas **sensitivity** represents the amount of output change per 1% change in RH.


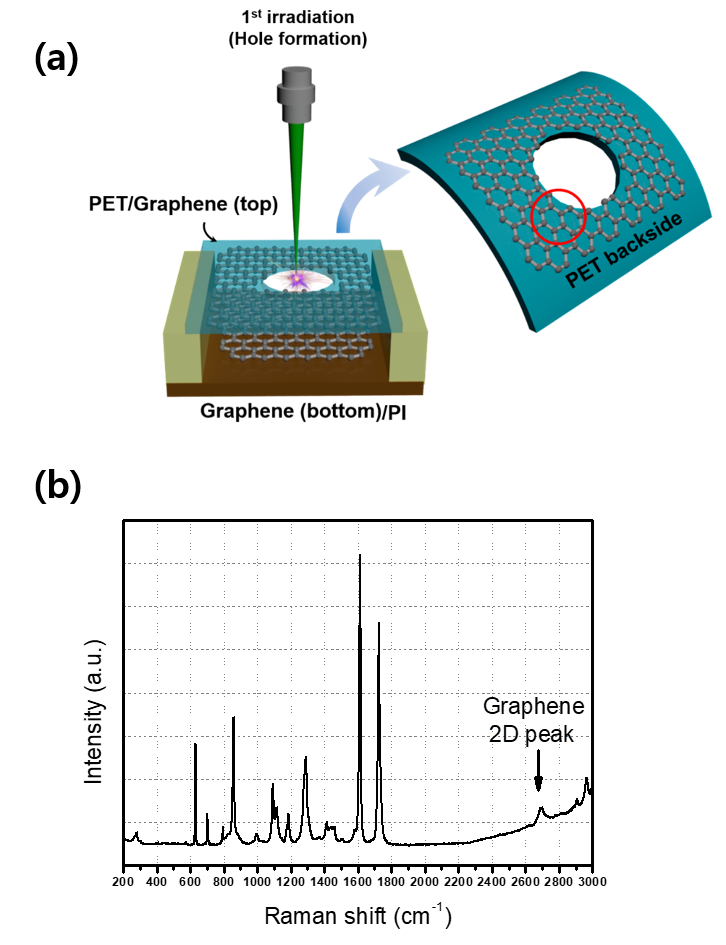


**Figure** **S1.** (a) Schematic of the first laser irradiation for hole formation in the PET film. (b) Raman peaks of the surrounding area after hole formation (in PET film), following single-pulse laser irradiation.


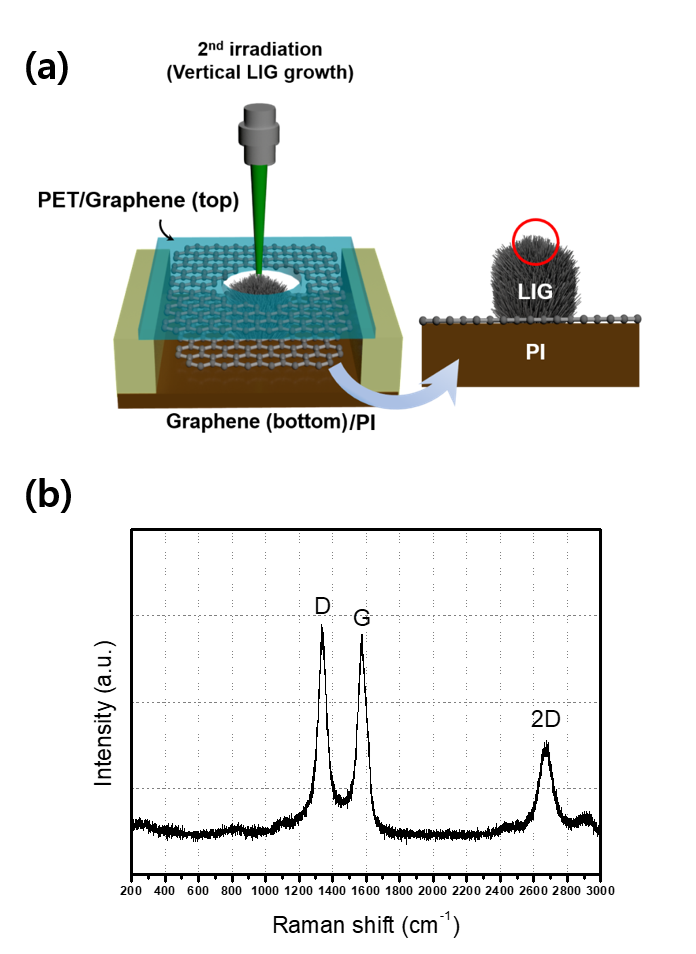


**Figure S2.** (a) Schematic of the second laser pulse used for LIG growth in the PET film. (b) Raman peaks of LIG grown by single-pulse laser irradiation.


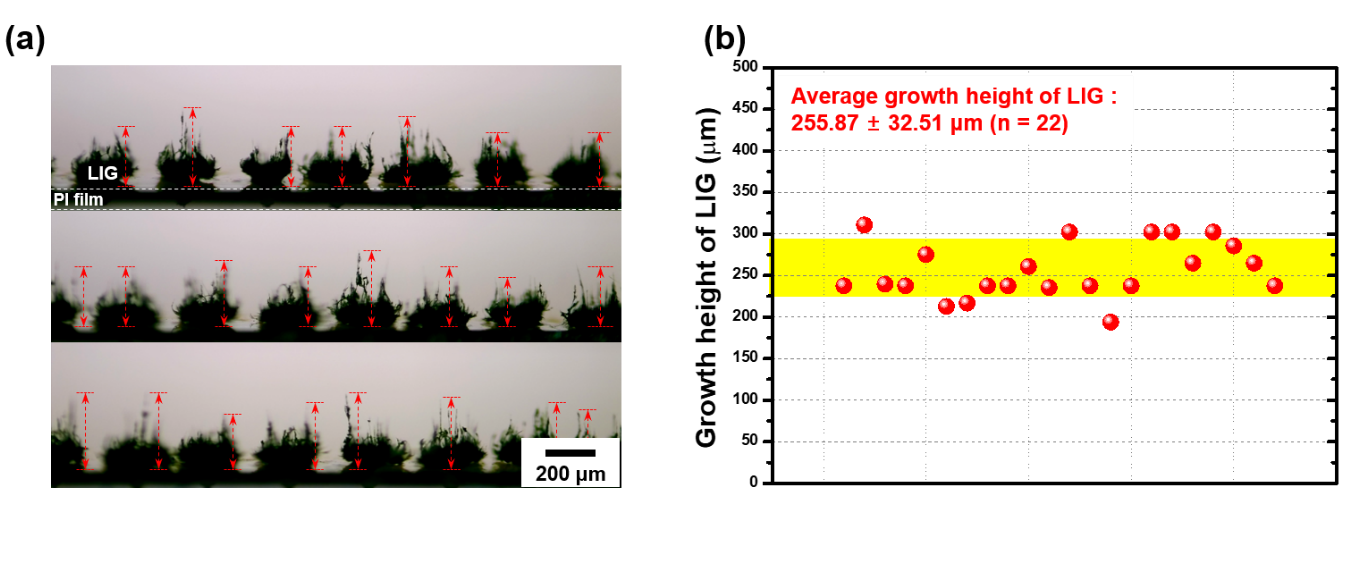


**Figure S3.** Statistical analysis of the vertical growth height of laser-induced graphene (LIG) structures. (a) Representative cross-sectional optical microscopy (OM) images of LIG structures formed on polyimide (PI) film, showing measured heights (n = 22). (b) Scatter plot of the measured LIG heights, with the average growth height determined to be 255.87 ± 32.51 μm (n = 22). The yellow band indicates the range of one standard deviation around the mean. (Fluence: 89.9 J/cm^2^, laser parameters: PPI = 130, and focus offset = 0 mm)


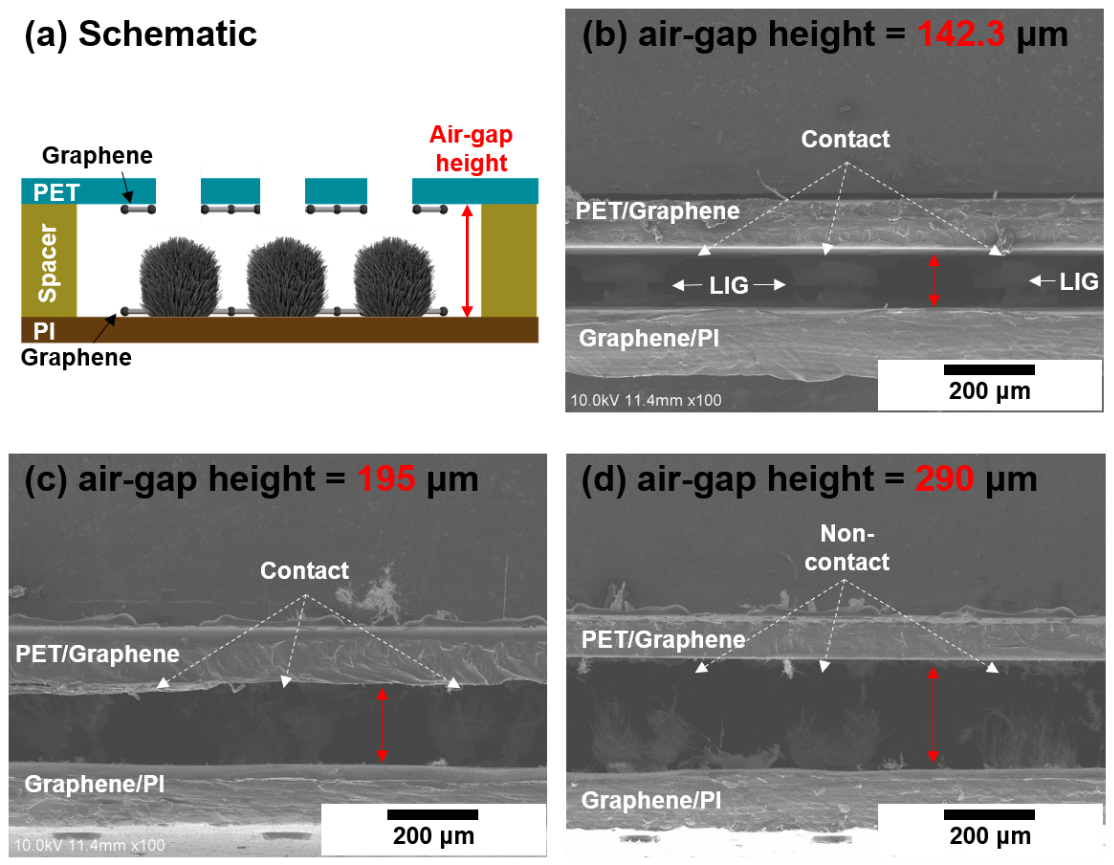


**Figure S4.** Cross-sectional SEM images of LIG growth under various air-gap height conditions. (a) Schematic of the vertical-contact sensor configuration with varying air-gap heights between the PET/graphene top electrode and PI/graphene bottom substrate. (b–d) SEM images of LIG grown at different air gaps: (b) 142.3 μm, (c) 195 μm, and (d) 290 μm. At air gaps less than 195 μm, the LIG structures consistently reached and made contact with the top electrode; however, at 290 μm, the LIG tips failed to bridge the gap. This indicates that the average LIG growth height remained at approximately 230 μm under the applied laser fluence. These results experimentally validated the operational threshold distinguishing the Contact and Remote Modes in the sensor design.


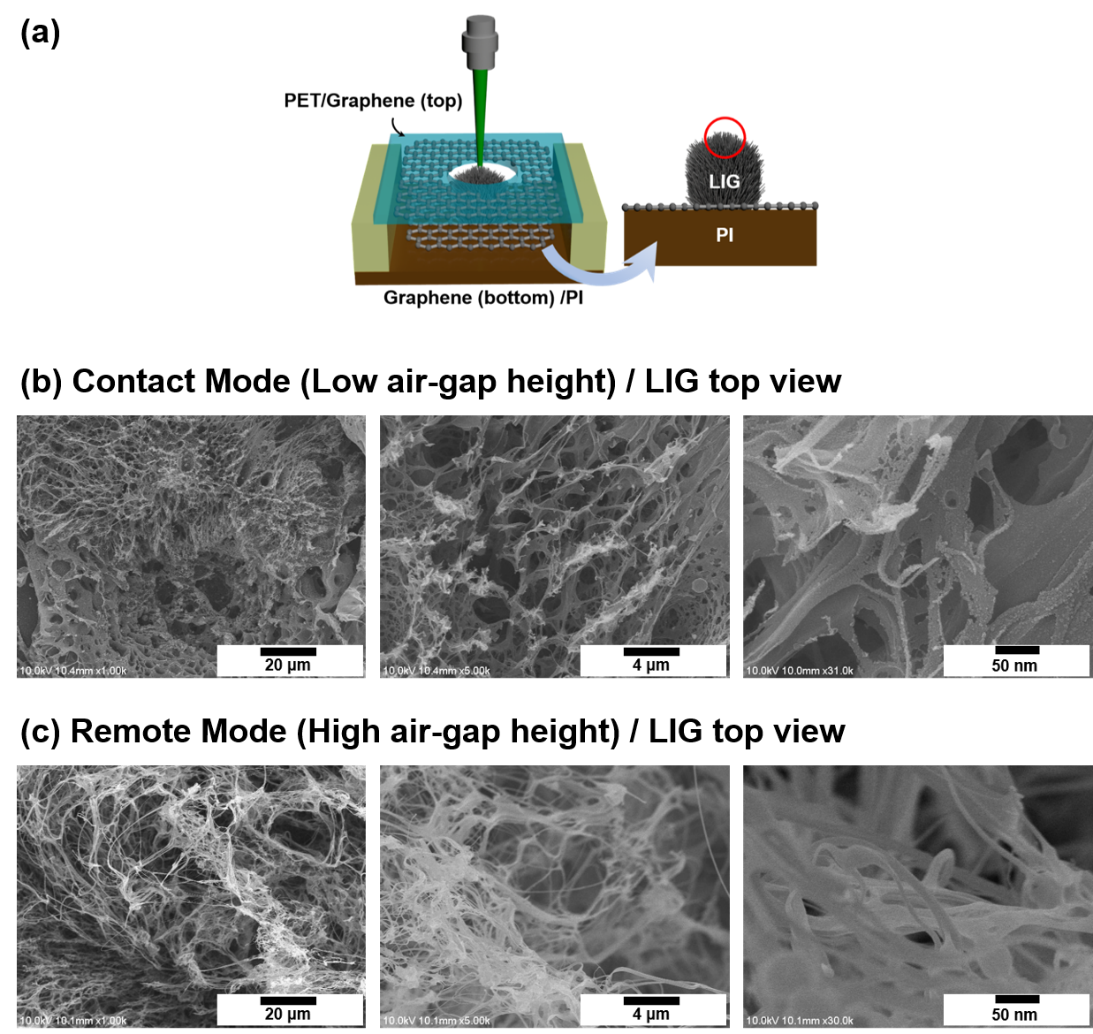


**Figure S5**. SEM images of LIG morphology in Contact and Remote Mode sensors. (a) Schematic of the PET/graphene layer being removed to expose the LIG structure underneath for direct SEM analysis. The red-circled area indicates the region analyzed. (b) SEM images of LIG in Contact Mode (air gap < LIG height), demonstrating a sheet-like morphology owing to constrained growth upon contact with the top electrode. (c) SEM images of LIG in Remote Mode (air gap > LIG height), exhibiting fibrous and thread-like structures, indicative of unconstrained vertical growth. These morphological differences confirm that the air-gap height directly influences LIG formation and structure.


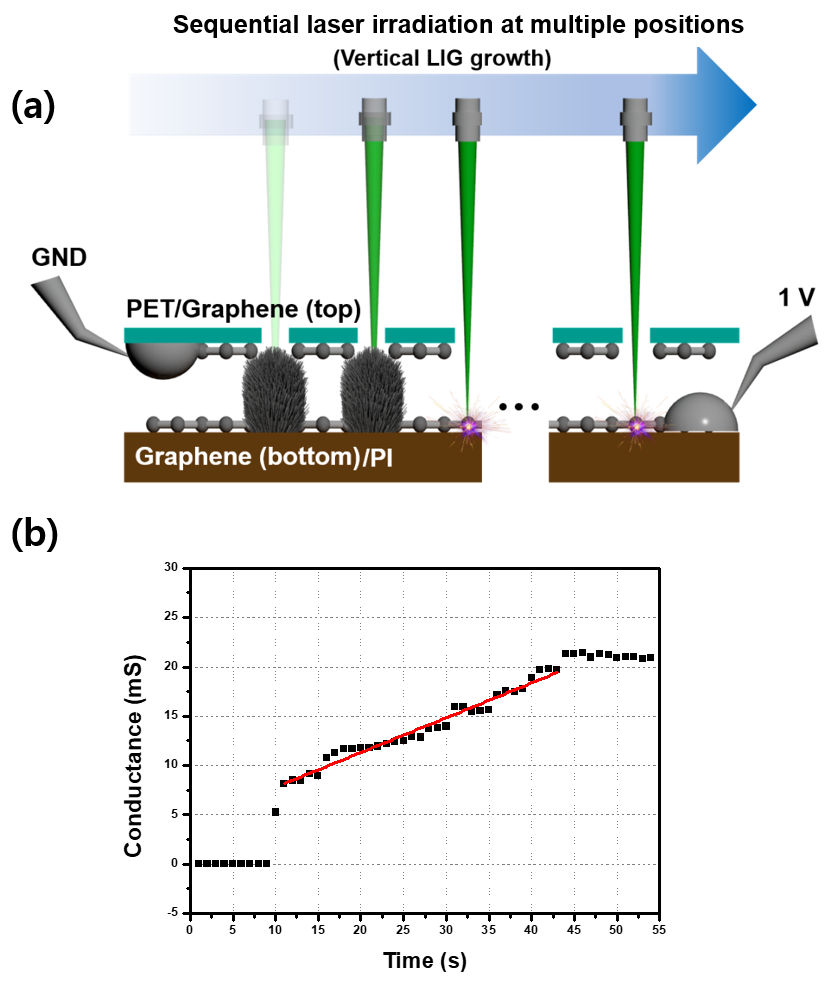


**Figure S6.** (a) Schematic of sequential laser irradiation at multiple positions. (b) Real-time changes in conductance with respect to the laser irradiation count. The conductance increases linearly with the number of LIG nanotips grown by single-pulse laser irradiation, demonstrating that LIG serves as a pathway for electron transport (air-gap height = 100 μm, laser fluence for hole formation and LIG growth = 89.9 J/cm², dot spacing = 0.2 cm).

**
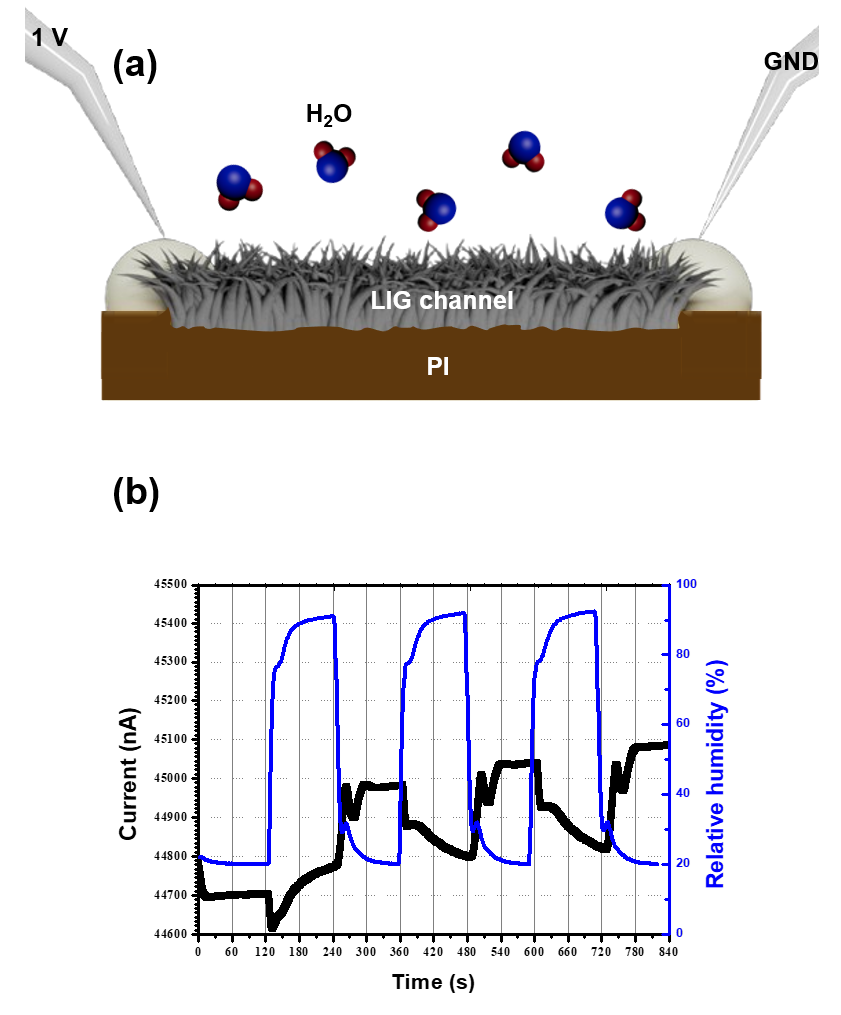
**

**Figure S7.** (a) Schematic of conventional LIG humidity sensor in lateral conduction path. (b) Current variation of the laser-induced graphene channel (20 mm × 30 mm bulk) as a function of humidity (laser parameters: Raster Mode, power = 55%, scan rate = 100%, PPI = 500, and focus offset = 0 mm)


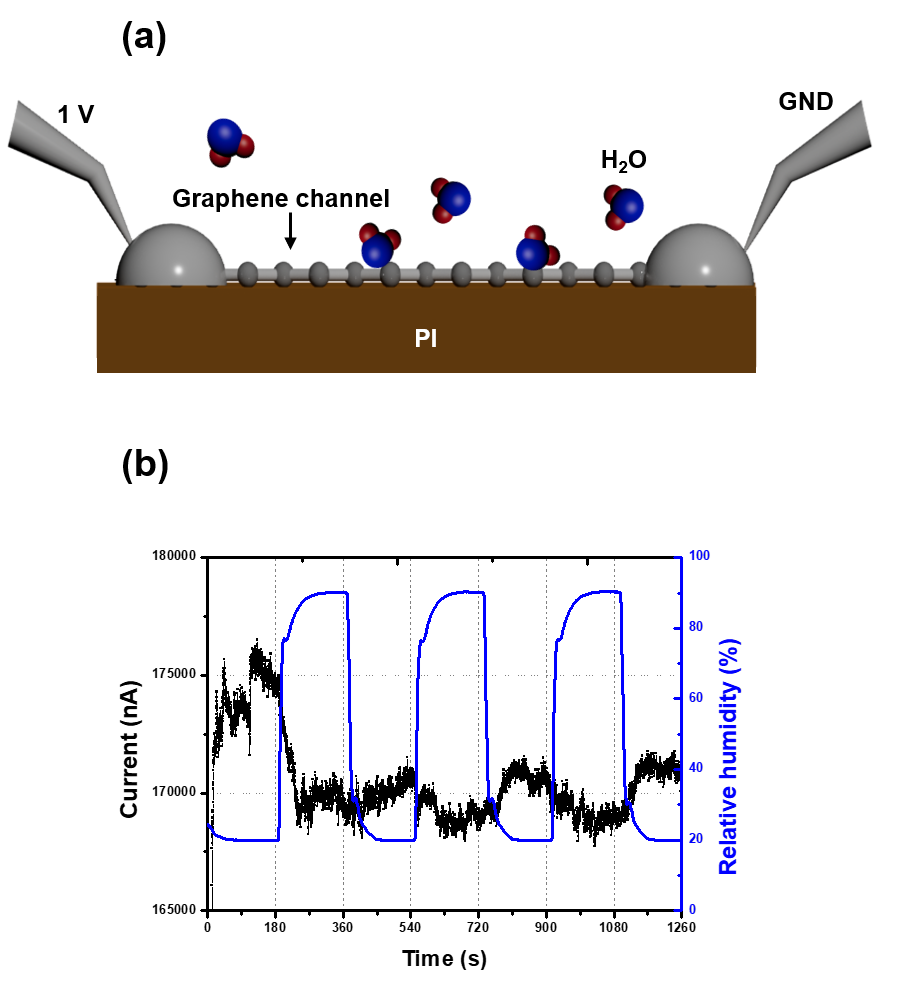


**Figure S8.** (a) Schematic of monolayer graphene channel. (b) Current variation of the graphene channel (20 mm × 30 mm) as a function of humidity. The CVD-grown monolayer graphene on Cu foil was transferred onto a PI film using the TRT method for electrical measurements.


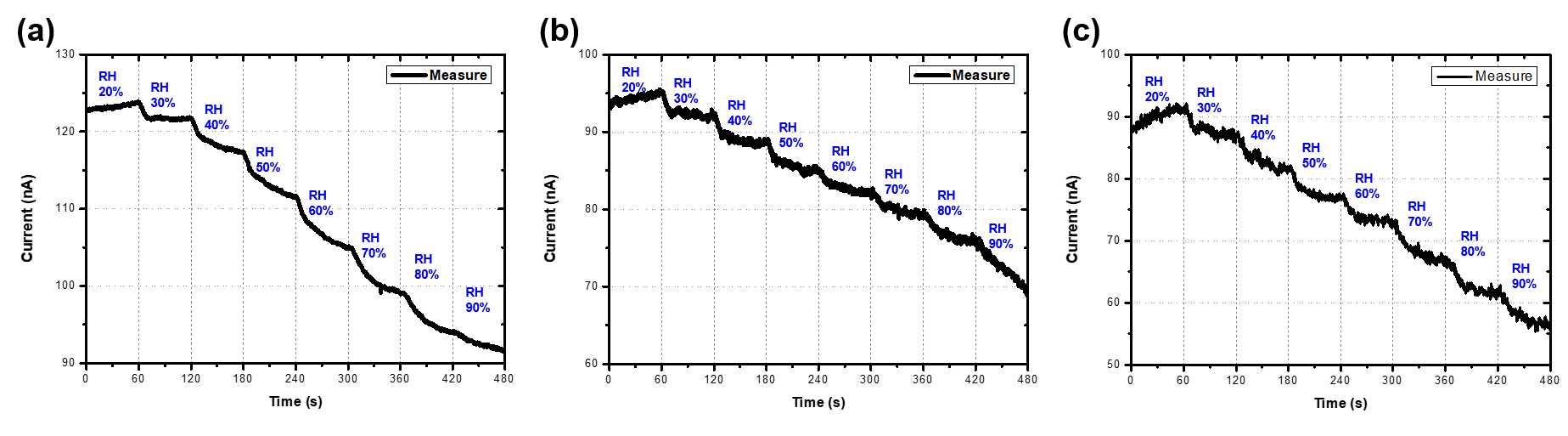


**Figure S9.** Raw current response data for three independently fabricated Contact Mode sensors measured under increasing RH from 20% to 90%. The continuous decrease in the current across all three samples demonstrates the excellent consistency and repeatability of the sensor performance. These raw data support the reproducibility of the sensor responses presented in Figure 3d of the main manuscript.


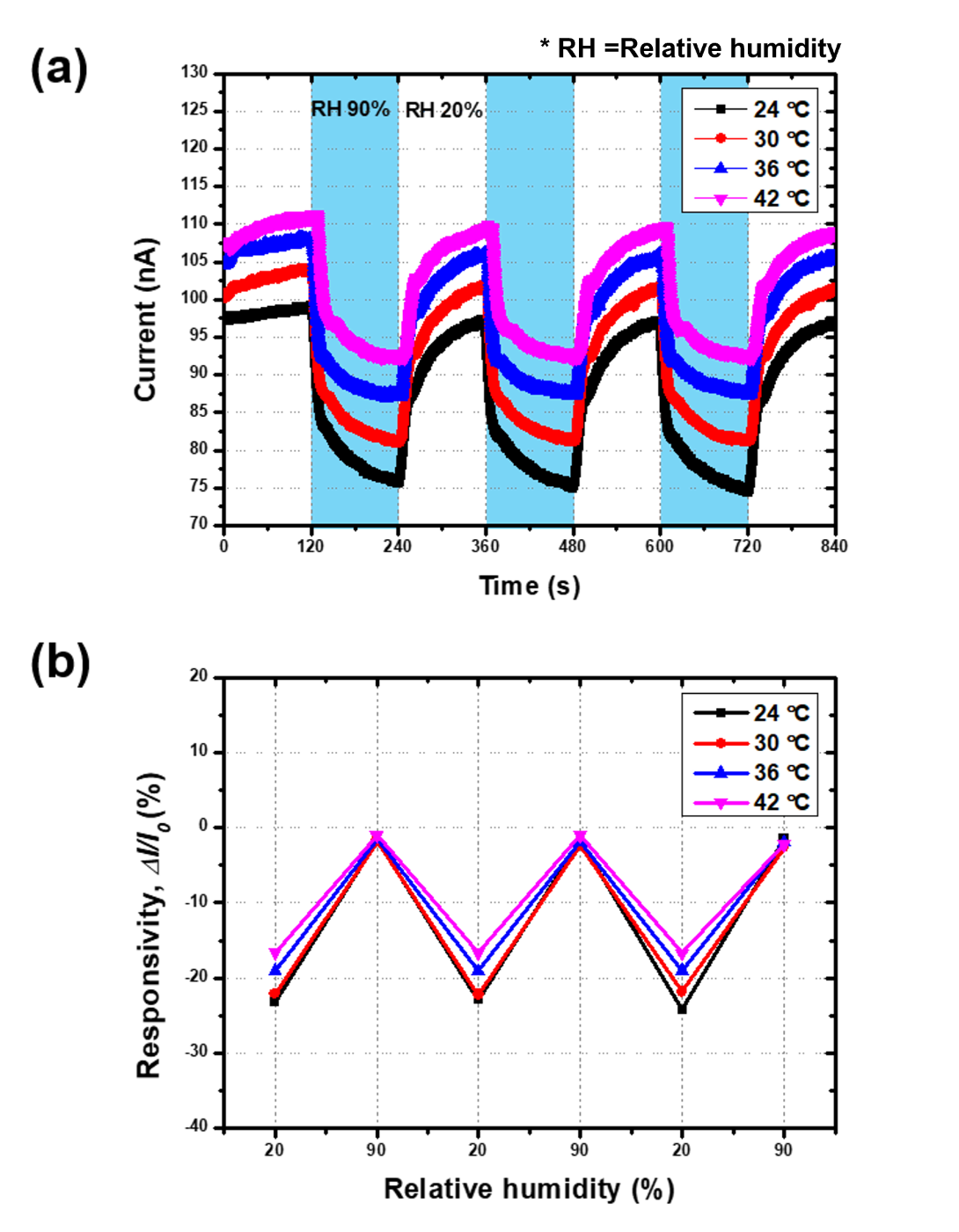


**Figure S10.** (a) Current measurement as a function of time at different temperatures (24, 30, 36, and 42 °C) while cyclically varying the RH between 20 and 90% in Contact Mode. (b) Responsivity (Δ*I*/*I*_0_ (%)) as a function of RH at different temperatures, showing stable responsivity despite temperature variations.

In Contact Mode, although the absolute current values varied with temperature changes, the rate of change (that is, responsivity) remained stable. Furthermore, the sensor maintained reliable operation during inhalation and exhalation at temperatures exceeding 40 °C, demonstrating its applicability in diverse environmental conditions.


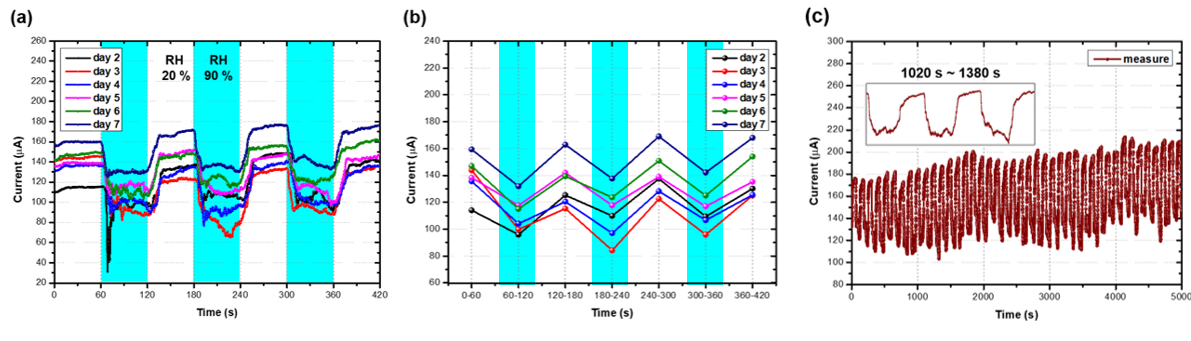


**Figure S11.** **(a)** Long-term stability of the Contact Mode sensor under sustained high-humidity conditions (RH 90%) for seven consecutive days. Each curve represents the current response measured on different days (days 2–7), showing consistent performance without significant signal degradation. **(b)** Extracted current values at RH = 20% and 90% over the 7-day test period, confirming the minimal drift and excellent stability of the sensing performance.
**(c)** Dynamic cycling test under alternating RH conditions (20% ↔ 90%) at 1-minute intervals for over 5,000 s. The sensor maintained clear and repeatable responses across all cycles without any deterioration, indicating strong durability under repetitive humidity exposures.

**
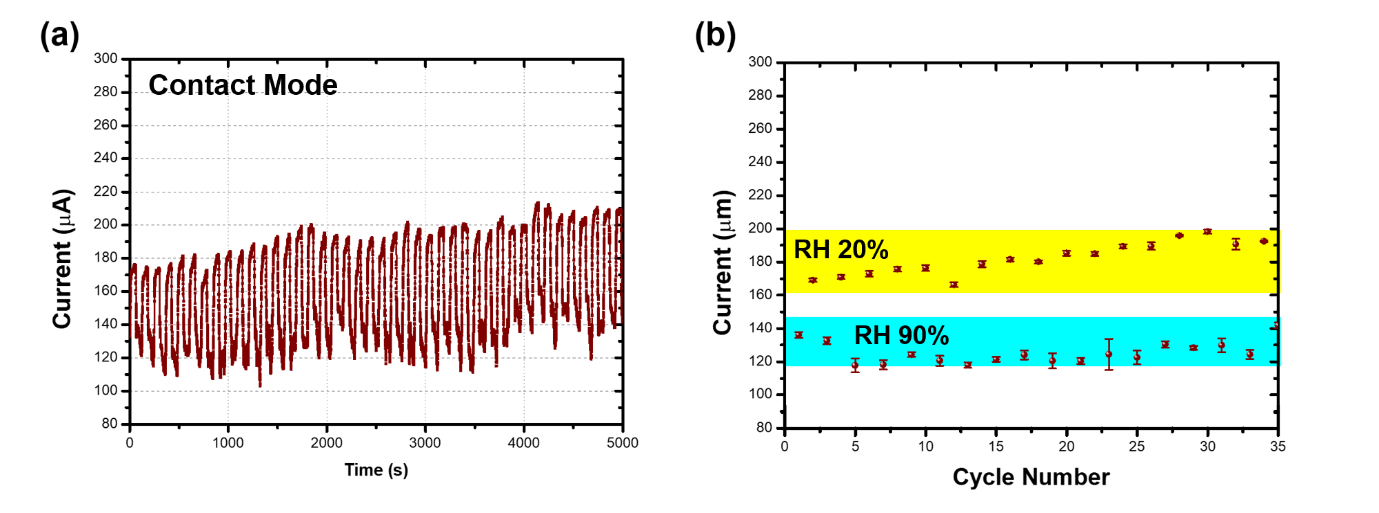
Figure S12.** Statistical analysis of the current response under stepwise RH variation for evaluating repeatability in Contact Mode. (a) Raw current response of the LIG-based humidity sensor under varying RH from 20% to 90% in 1-minute intervals over 5,000 s. (b) Average current values with standard deviation at each RH step, calculated from a 20-second stable region within each interval.


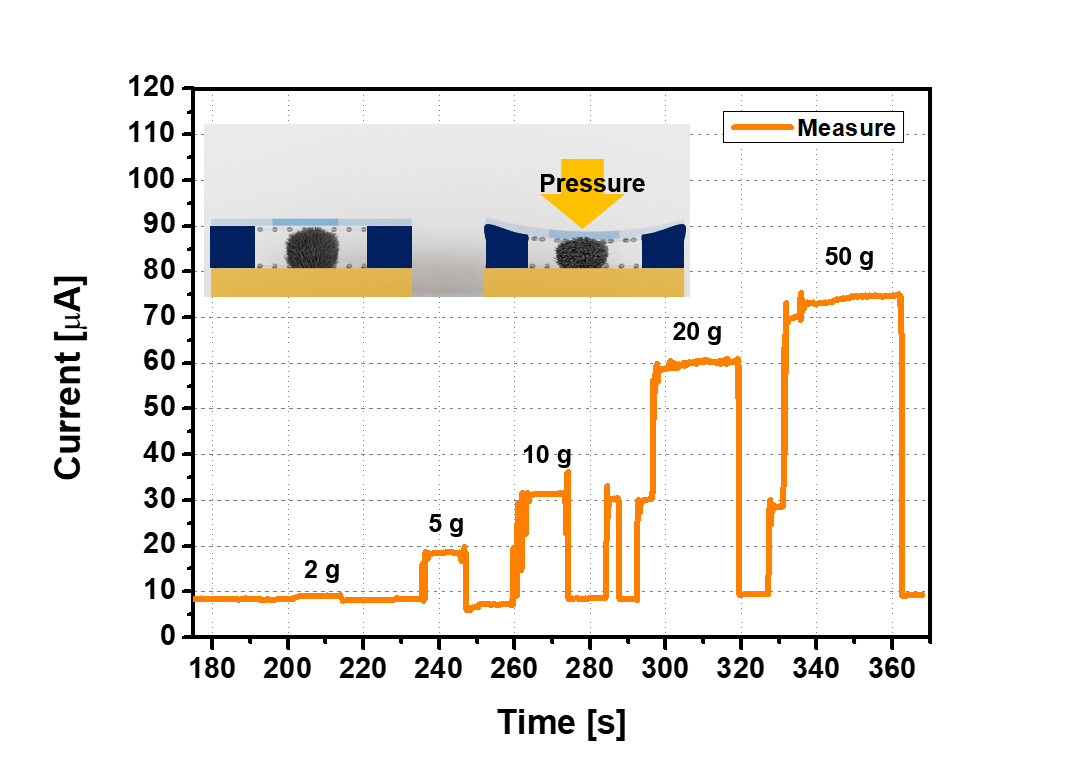


**Figure S13.** Self-recovery behavior under pressure in Contact Mode. The graph shows the measured current response during repeated application and removal of external pressure. When pressure is applied, the contact area of the sensor increases, resulting in an increase in current level. However, once the pressure is released, the current reliably returns to its original baseline, demonstrating stable sensor operation.

**
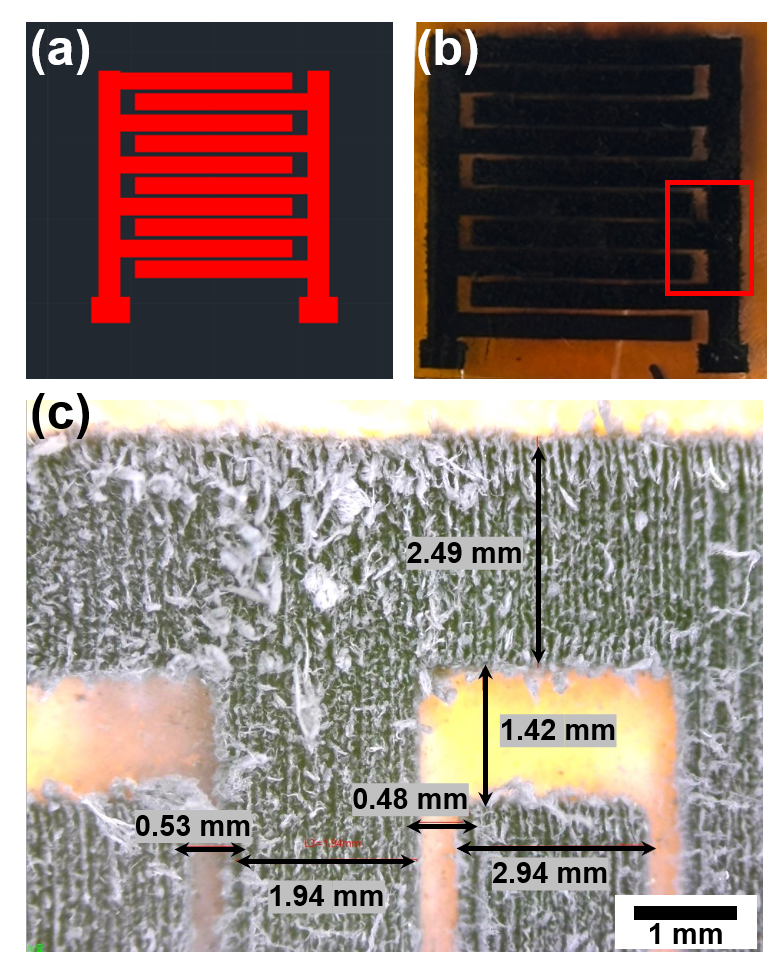
**

**Figure S14.** Optical microscope (OM) image of the IDE pattern used for comparison and its laser parameters: Raster Mode, power = 55%, scan rate = 100%, PPI = 500, and focus offset = 0 mm. (a) AutoCAD software (Autodesk), (b) OM, and (c) magnified OM images.

**
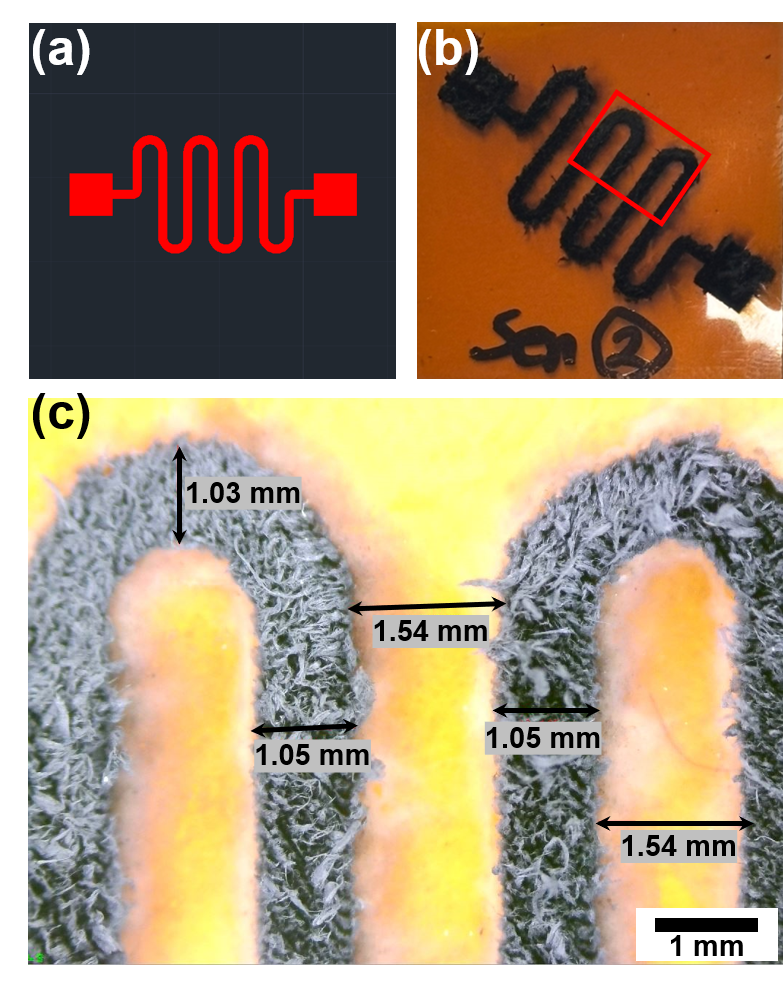
**

**Figure S15.** OM image of the serpentine pattern used for comparison and its laser parameters: Raster Mode, power = 55%, scan rate = 100%, PPI = 500, and focus offset = 0 mm. (a) AutoCAD (computer-aided design) software image, (b) OM, and (c) magnified OM images.


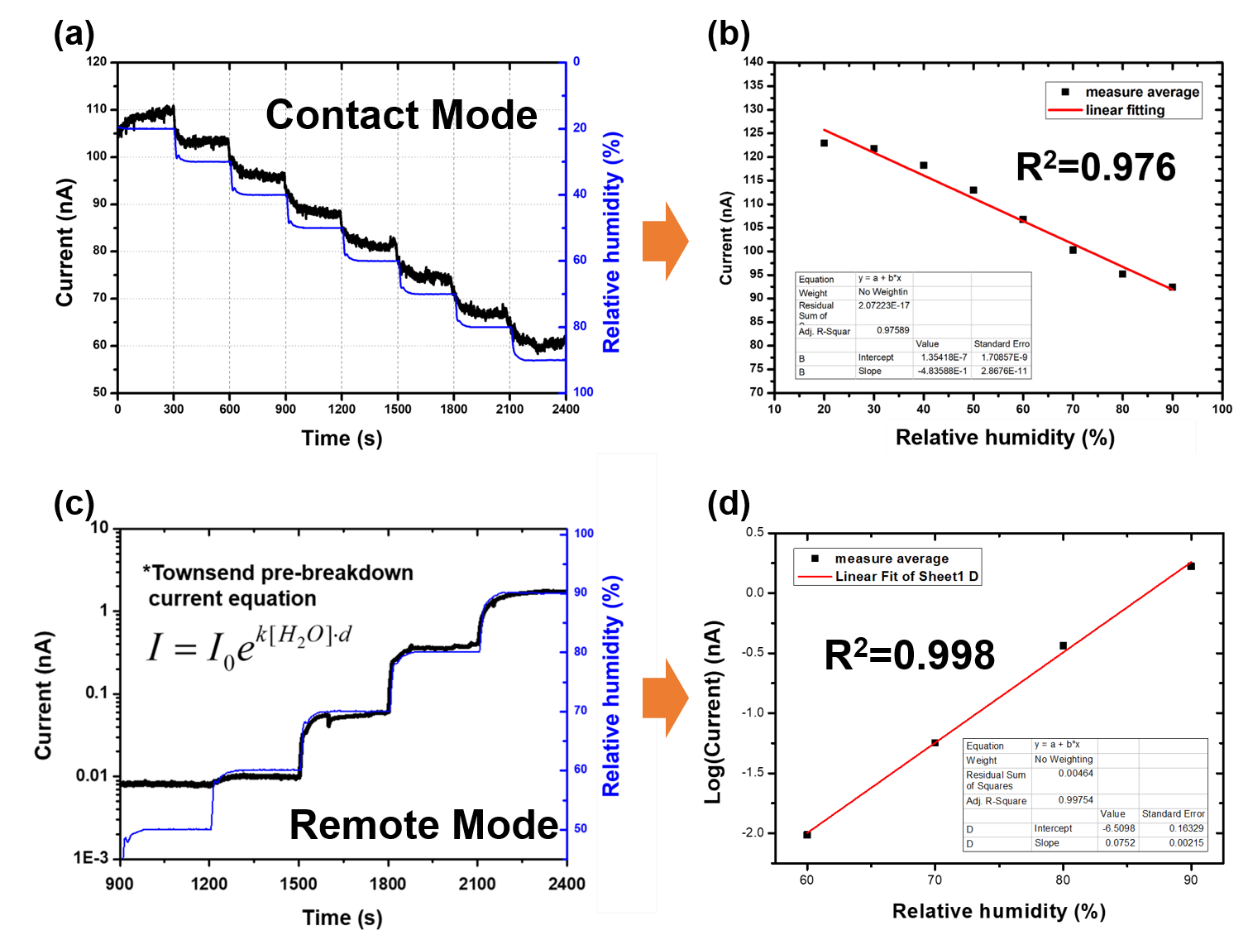


**Figure S16.** Evaluation of the Linear Response of Contact and Remote Mode Sensors to RH (a) Raw current response of the Contact Mode sensor under stepwise RH increase (20% → 90%), showing a linear decrease in current. (b) Linear fitting of Contact Mode responsivity versus RH data, yielding a high coefficient of determination (R² = 0.9759), confirming its linear response. The responsivity values were calculated using the average current measured after the RH level stabilized at each step. (c) Raw current response of the Remote Mode sensor, exhibiting exponential behavior in accordance with the Townsend pre-breakdown current model. (d) Linear fitting of log(current) versus RH data for the Remote Mode, demonstrating a strong log-linear response with R² = 0.9975. The responsivity values were calculated using the average current measured after the RH level stabilized at each step.


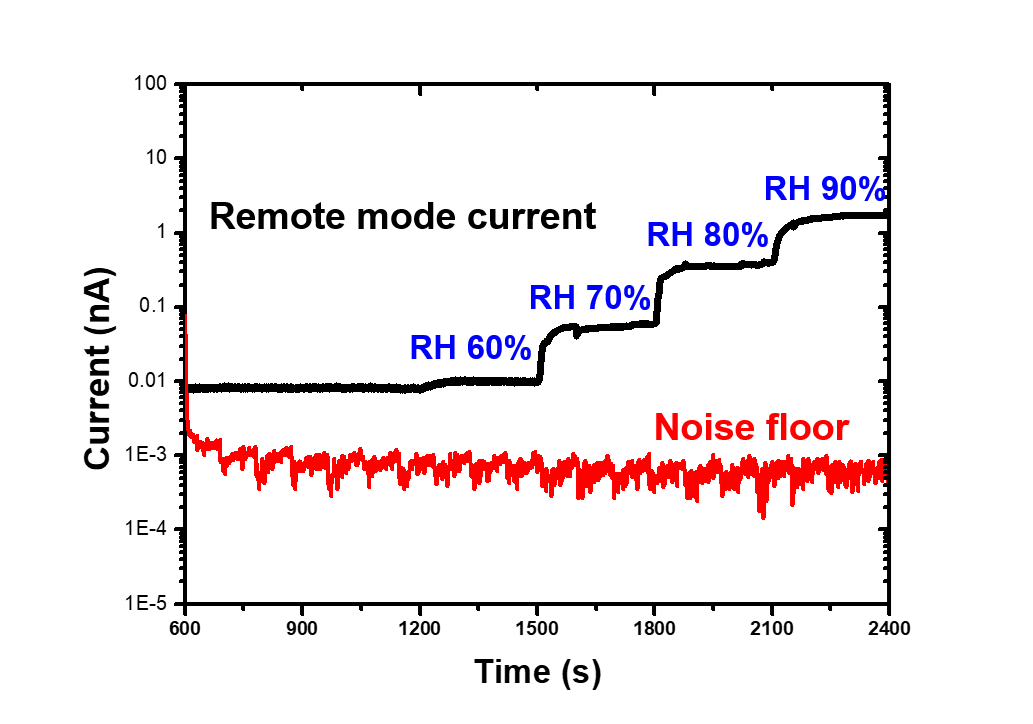


**Figure S17**. Comparison between the sensor current (black) in Remote Mode and the open-circuit background current (red) under identical humidity conditions. Even under low humidity (RH < 60%), the sensor current remained slightly above the noise floor, and an exponential increase was observed as the RH exceeded 60%.


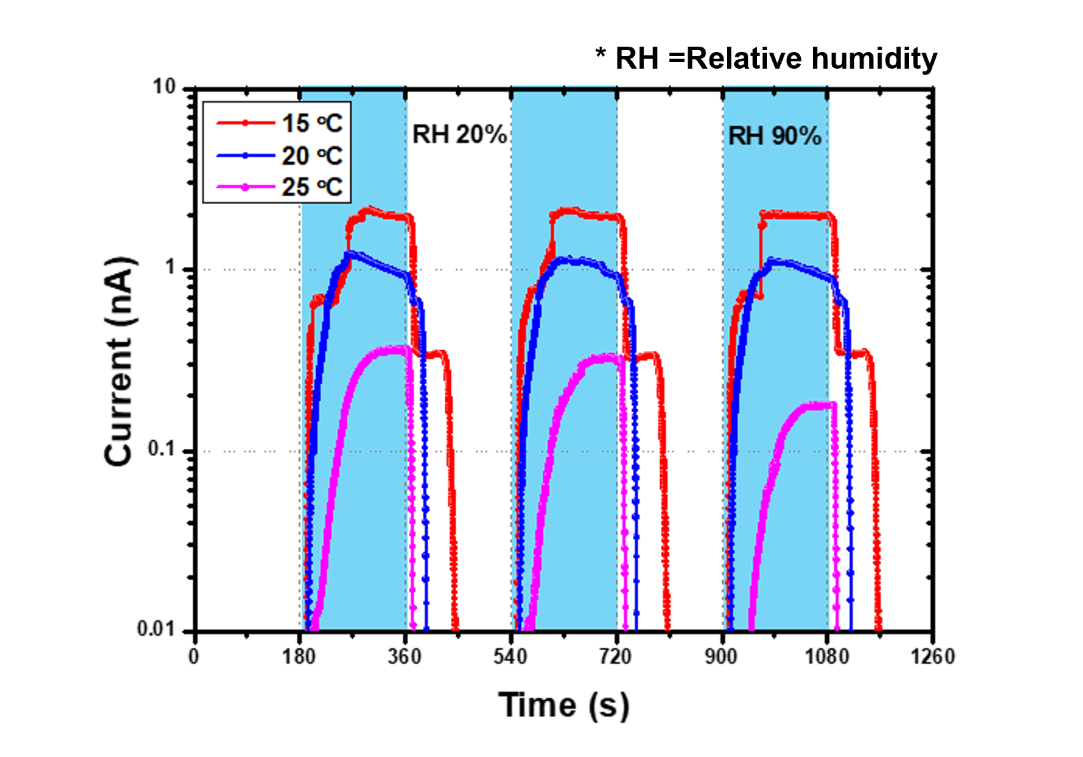


**Figure S18.** Current measurement as a function of temperature (15, 20, and 25 °C) while cyclically varying RH between 20 and 90% in Remote Mode.

In Remote Mode, the sensor exhibited clear ON/OFF behavior within the temperature range of 15–25 °C. As the temperature increased, the absolute current decreased, which can be attributed to the thermodynamic relationship between temperature and humidity. With increasing temperature, the saturation vapor pressure *P _sat_* also increased, following the empirical expression:

$P_{sat}=P_{0}\exp\left( -\frac{L}{RT} \right)$, (S8)

where *P_0_*​ denotes the reference pressure, *L* represents the latent heat of vaporization, *R* indicates the specific gas constant for water vapor, and *T* signifies the absolute temperature in Kelvin.

Because *RH* is defined as the ratio of the partial pressure of water vapor

*P _vaper_*​ to the saturation vapor pressure *P _sat_*, we have

$RH(\%)=\left( \frac{P_{vaper}}{P_{sat}} \right)\times100$. (S9)

In a semi-enclosed chamber where wet gas is continuously introduced and vented, an increase in temperature elevates *P _sat_*​, leading to decreased *RH* because *P_vaper_*​ ​may not increase at the same rate. Consequently, the number of water molecules present in the chamber decreases, thereby lowering the probability of impact ionization and resulting in relatively lower current amplification. Notably, during exhalation, although the temperature increased, the sensor consistently remained in the ON state, whereas during inhalation, when the temperature decreased, the sensor switched to the OFF state. This behavior suggests that the sensor does not merely respond to temperature variations but is primarily sensitive to humidity. Therefore, the observed decrease in current with increasing temperature is consistent with the humidity-dependent mechanism of the sensor. These findings support the conclusion that LIG nanotip-based sensors enable stable and accurate humidity detection over a wide temperature range. This demonstrates their suitability for real-world applications, such as continuous respiratory monitoring in dynamically changing environments or during physical activity.


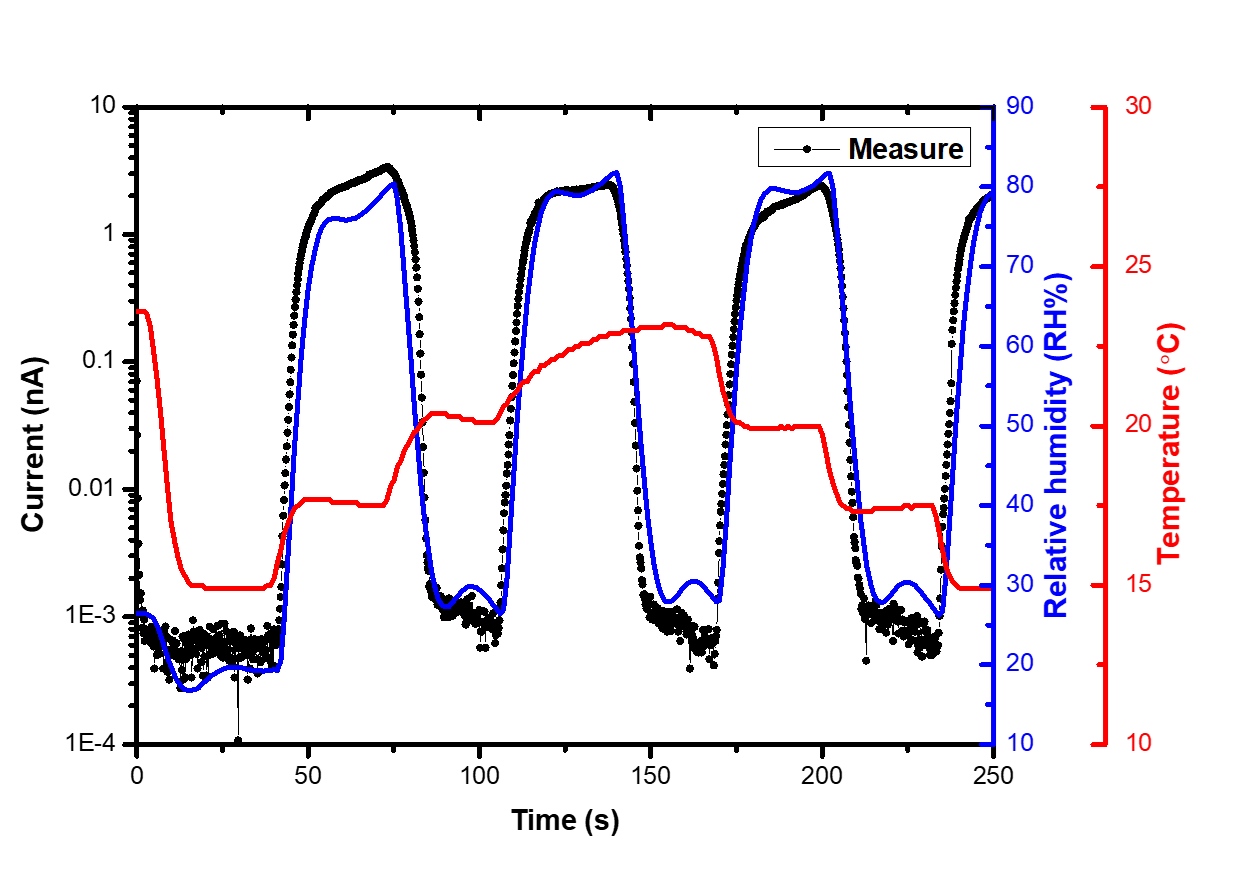


**Figure S19.** Real-time response of the LIG sensor under varying environmental conditions. Although both temperature (red) and RH (blue) varied simultaneously, the measured current (black) closely followed the RH profile, indicating that the sensor's response was predominantly governed by humidity rather than temperature.**
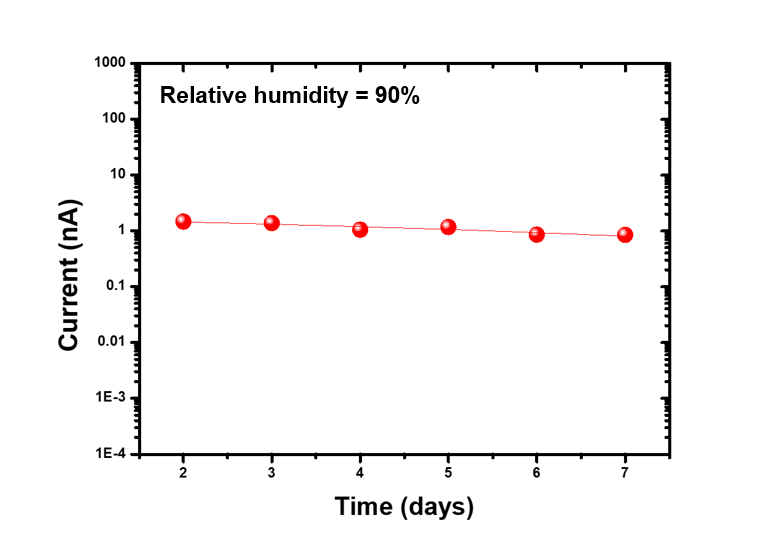
**

**Figure S20.** Long-term stability test of the Remote Mode sensor for seven days.

The LIG nanotip-based humidity sensor detects humidity by inducing pre-breakdown current amplification via field ionization, thereby eliminating the need for direct physical contact with water molecules. Because this process occurs without direct interaction between the LIG nanotip and top graphene layer, the sensor maintains a highly stable response over time without degradation or malfunction. As confirmed by a 7-day stability test conducted under controlled RH and temperature conditions (90% RH, 20 °C), the sensor demonstrated consistent performance with minimal signal drift.

Long-term exposure to varying humidity levels and temperature fluctuations should be investigated further to ensure extended durability in practical applications. Additionally, further long-term testing over extended periods is necessary to fully validate the robustness of the sensor under real-world conditions. These findings establish the durability and reliability of the sensor, making it highly suitable for long-term environmental and industrial applications.

**
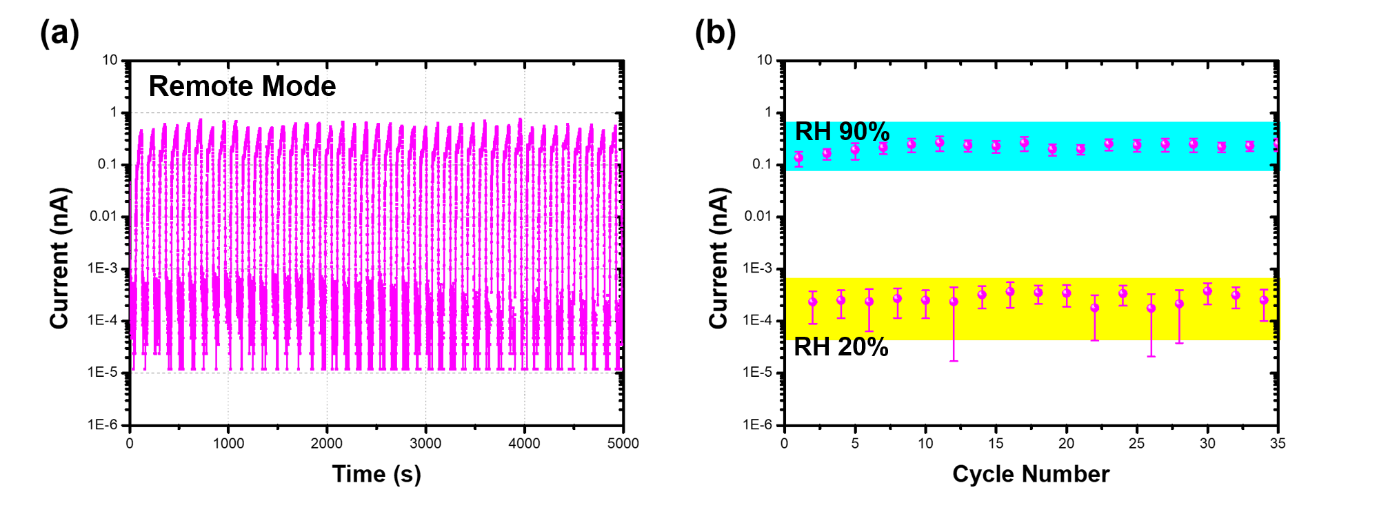
** **Figure S21.** Statistical analysis of current response under stepwise RH variation for evaluating repeatability in Remote Mode. (a) Raw current response of the LIG-based humidity sensor under varying RH from 20% to 90% in 1-minute intervals over 5,000 s. (b) Average current values with standard deviation at each RH step, calculated from a 20-second stable region within each interval.


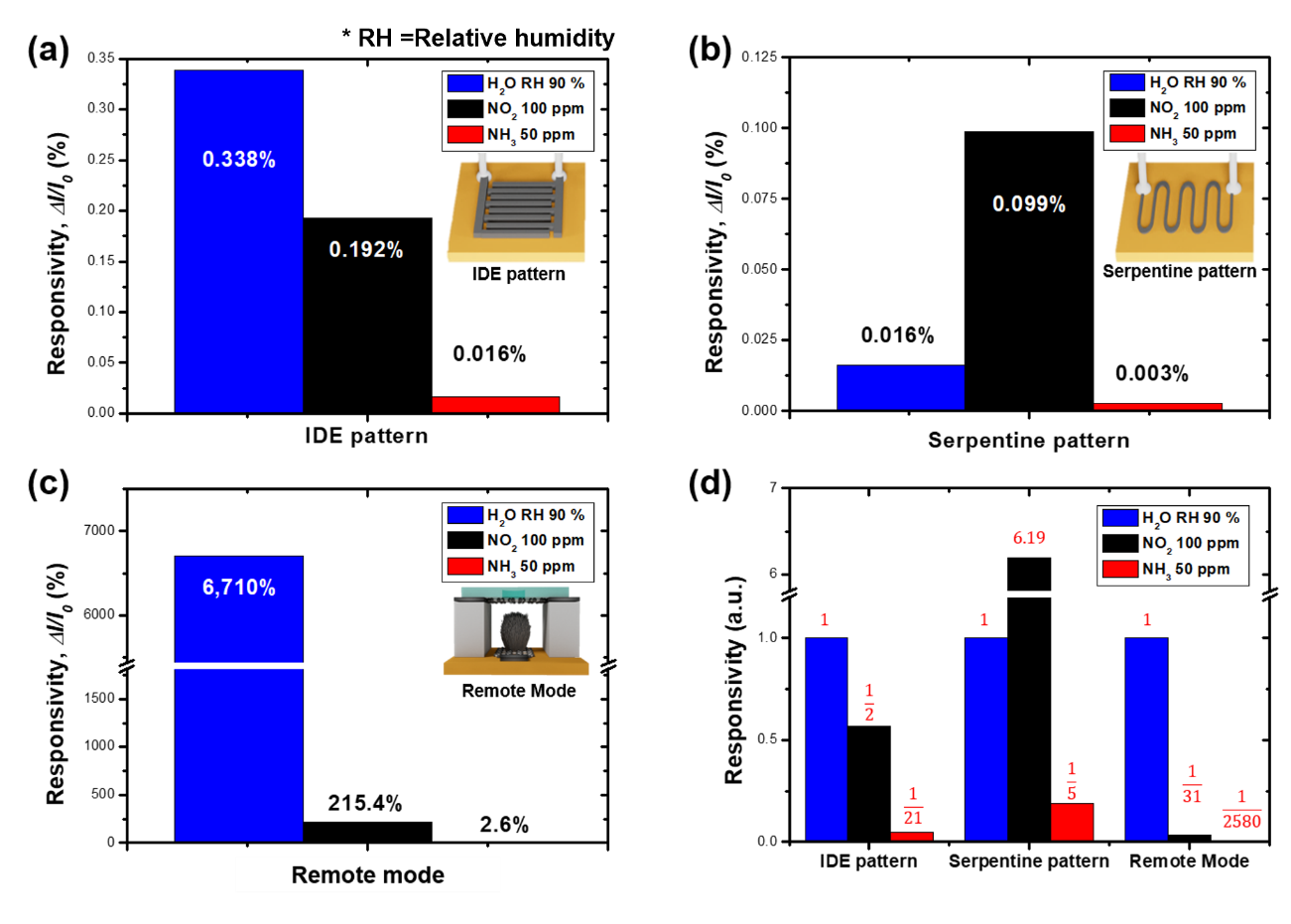


**Figure S22.** Selectivity measurement of Remote Mode for different gases. (a–c) Schematics and corresponding responsivity of IDE-patterned, serpentine-patterned, and Remote Mode sensors toward H₂O (RH 90%), NO_2_ (100 ppm), and NH_3_ (50 ppm), respectively. (d) Responsivity comparison for each gas type across all three sensor types.

In this study, we evaluated the selectivity characteristics of LIG-based humidity sensors fabricated using three different electrode configurations: IDE, serpentine, and Remote Mode. The experiments were conducted in a sealed chamber equipped for current measurements, with precise control of gas flow conditions. As shown in Figure S11a–c, each sensor exhibited distinct responsivity trends when exposed to H₂O (RH 90%), NO_2_ (100 ppm), and NH_3_ (50 ppm). Specifically, the IDE-patterned sensor exhibited responsivities of 0.338% for H_2_O, 0.192% for NO_2_, and 0.016% for NH_3_, whereas the serpentine-patterned sensor exhibited responsivities of 0.016% for H_2_O, 0.099% for NO_2_, and 0.003% for NH_3_ (Figure S11a–b). These results indicate that both conventional sensors lacked sufficient humidity selectivity, as their responses to H_2_O were comparable to or even lower than those for NO_2_ or NH_3_.

By contrast, the Remote Mode sensor developed in this study demonstrated a significantly enhanced response of 6,710% to H_2_O, 215% to NO_2_, and 3% to NH_3_ (Figure S11c). Selectivity ratio analysis (Figure S11d) further confirmed that the sensor response to H_2_O was approximately 31 times higher than that of NO_2_ and 2,580 times higher than that of NH₃. This remarkable selectivity is attributed to the enhanced interaction between gas molecules and the LIG surface, where molecules such as H_2_O—which have high polarity and low ionization energy—exhibit stronger responses under an electric field. Given that the dielectric constant of H_2_O (approximately 80) is significantly higher than that of NO₂ (approximately 2.4), and its polarity is also much stronger, electron emission is more effectively facilitated under the local electric field [S1]. These findings confirm that the proposed Remote Mode sensor is a highly optimized and humidity-selective platform, offering strong potential for precise environmental monitoring and industrial humidity control systems.


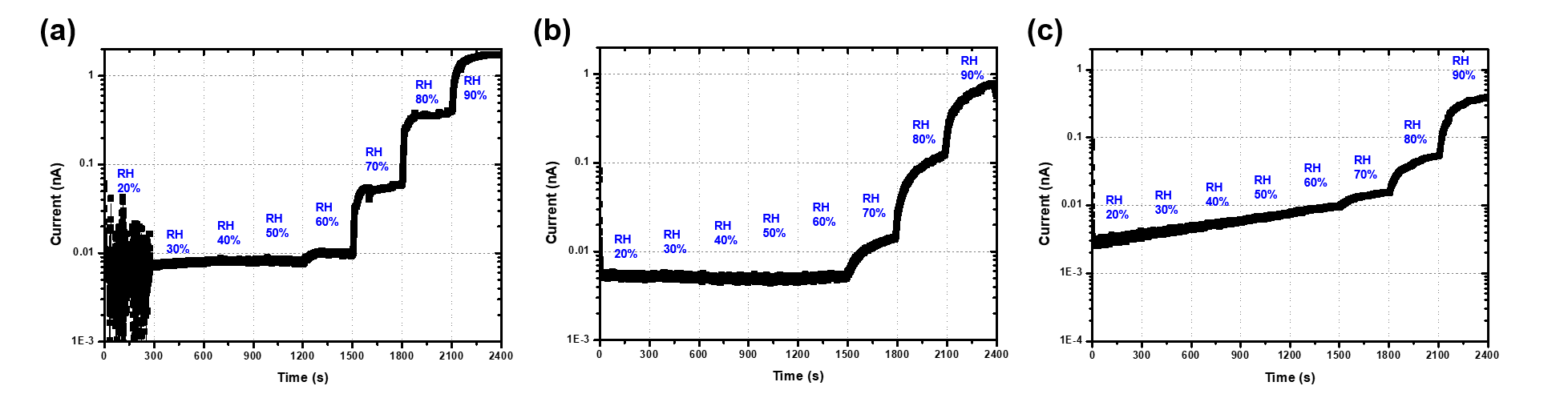
**Figure S23.** Raw current response data for three independently fabricated Remote Mode sensors under increasing RH from 20% to 90%. All samples exhibited consistent exponential increases in the current, quantitatively supporting the results shown in Figure 4d. The calculated responsivities of the three sensors were 13,598%, 6,711%, and 6,350%, respectively.

**Electric-Field Enhancement Effect by Controlling the Gap Between LIG Nanotips** **and Top Electrode**

Precise control of the gap (*d*) between the LIG nanotips and the top graphene layer is essential for optimizing sensor performance, as it directly affects the electric-field strength and ionization efficiency. The Townsend equation discussed in the Remote Mode analysis assumes a fixed electrode gap (*d*) based on the already grown LIG nanotip structure. This assumption might lead to the misconception that increasing the electrode gap (*d*) will continuously increase the current, whereas, in reality, a larger gap reduces the local electric field, potentially limiting the ionization efficiency. However, to establish a more accurate current amplification model, the effect of the electric field (*E*) on the Townsend coefficient (α) must be considered in the model. The general expression for current in Townsend discharge [S2] is given by

$I={I_{0}}^{\alpha d}$. (S10)

Here, the Townsend coefficient (*α*) is a function of the electric field and follows the relationship:

$\alpha=Ae^{-B/E}$^,^ (S11)

where *A* represents a material-dependent constant related to the probability of ionization by electron impact, and *B* indicates a field-dependent constant that determines how rapidly the ionization coefficient decreases as the electric field weakens. *E* is the local electric-field strength, which is determined by the applied voltage and electrode gap. Substituting this into the current equation results in

$I=I_{0}e^{Ae^{-B/E}\cdot d}.$ (S12)

Additionally, *E* is inversely proportional to the electrode gap (*d*) and can be expressed as

*E*=*V*/*d.* (S13)

To validate this effect, finite element method simulations were conducted using COMSOL Multiphysics to analyze the variation of *E* with *d*. The simulation parameters are listed in Table S1. A 1 V bias was applied to the bottom graphene layer, and the resulting electric field was evaluated. The simulation, based on 30 representative nanotips, showed that when *d* < 5 μm, *E* exceeded the ionization threshold, enabling sustained field ionization (**Figure 4e**). As *d* decreases further, *E* increases exponentially, thereby enhancing the ionization. However, real-world sensors involve multiple nanotips, which collectively enhance *E* beyond the simulated values, thereby improving the ionization efficiency [S3]. When *d* is less than approximately 5 μm, the sensor operates in Remote Mode, whereas when *d* becomes less than 0, it transitions into Contact Mode, where direct charge transport dominates

Precise control over the growth height of LIG nanotips is essential for maintaining consistent sensor performance. However, variations in microscale fabrication present challenges in ensuring uniform responsivity (**Figure 4d**). Future research should focus on optimizing the growth height, nanotip density, and structural uniformity to improve the reproducibility and reliability of the sensors.

**
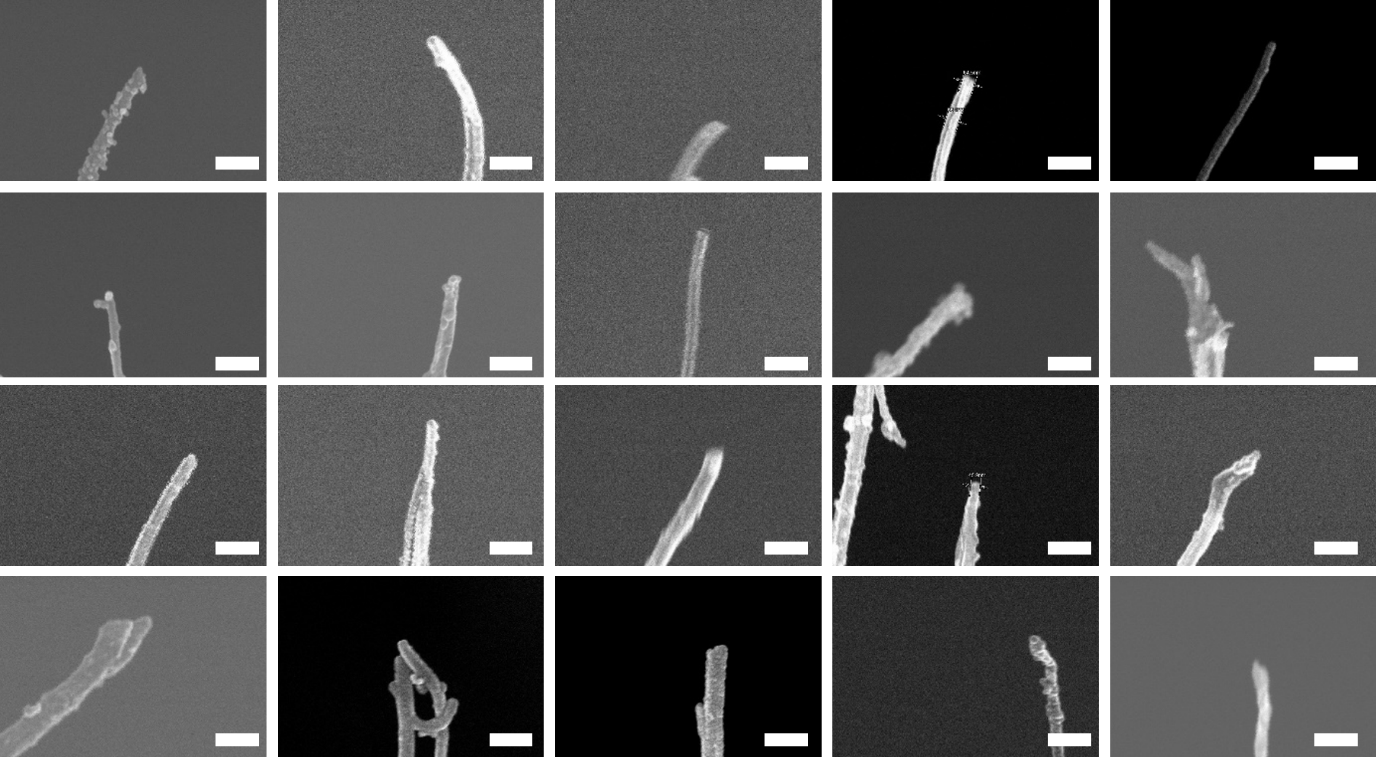
**

**Figure S24.** SEM images of actual LIG nanotips used in COMSOL simulation to determine nanotip shape. Scale bar = 200 nm.

**Table S1.** Parameters used for electric-field simulation (COMSOL).

| **Parameter** | **Value** |
| --- | --- |
| RH | 90% |
| Temperature | 20 °C |
| Air Permittivity | 1.01 [S4] |
| Air Conductivity | 10^−12^–10^−11^ S/m [S5] |
| LIG Conductivity | 1×10^3^–10^4^ S/m [S6] |
| LIG Permittivity | 3–5 |
| Substrate Permittivity | 5–11.7 |
| Graphene Work Function (Φ) | 4.3–4.7 eV [S7] |
| Graphene Conductivity (σ) | 10^6^ S/m [S8] |
| Graphene Permittivity (ε) | 1 |
| Graphene Thickness (h) | 0.34 nm |

**
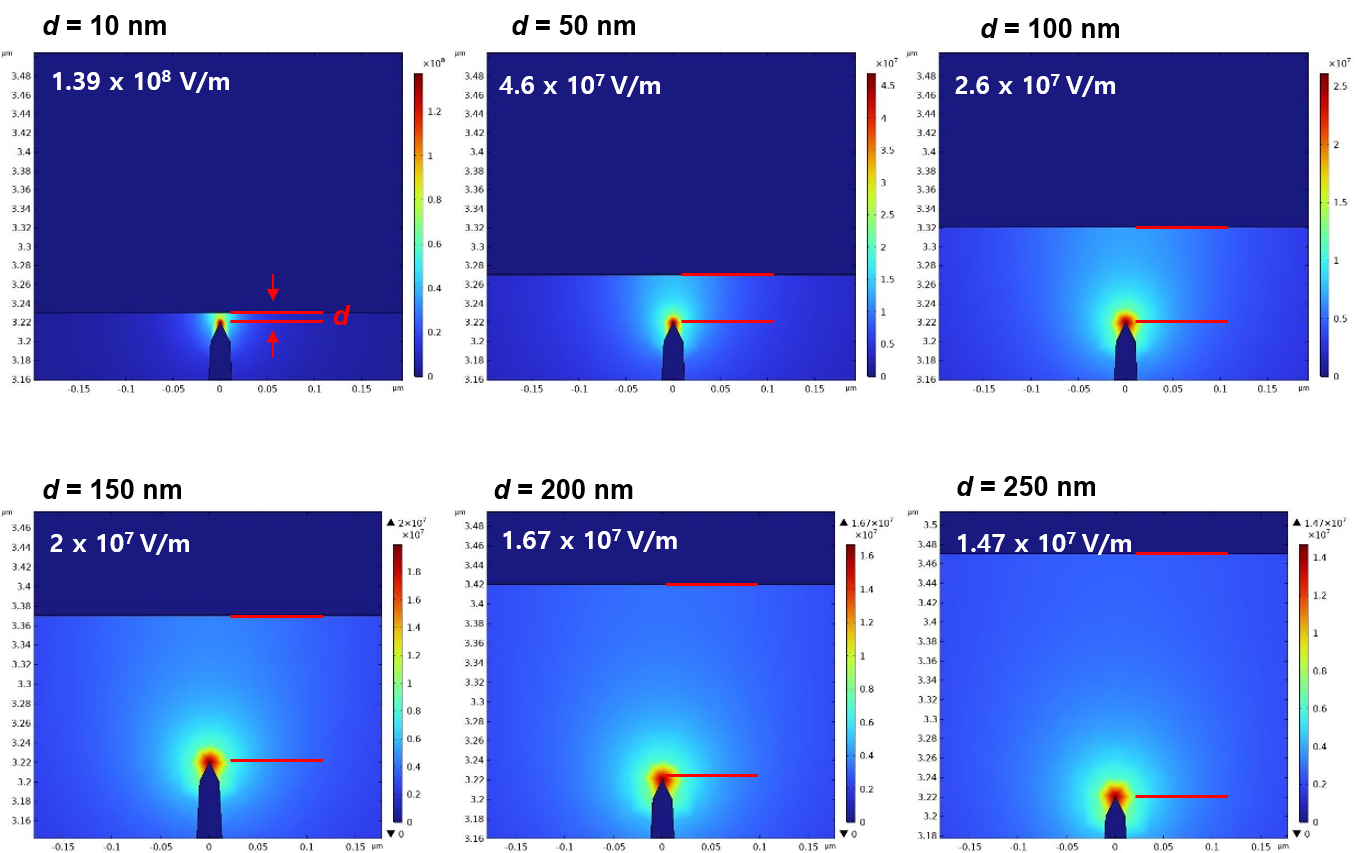
**

**Figure S25.** Electric-field simulation (COMSOL) based on the distance *d* between the top graphene electrode and LIG nanotip.


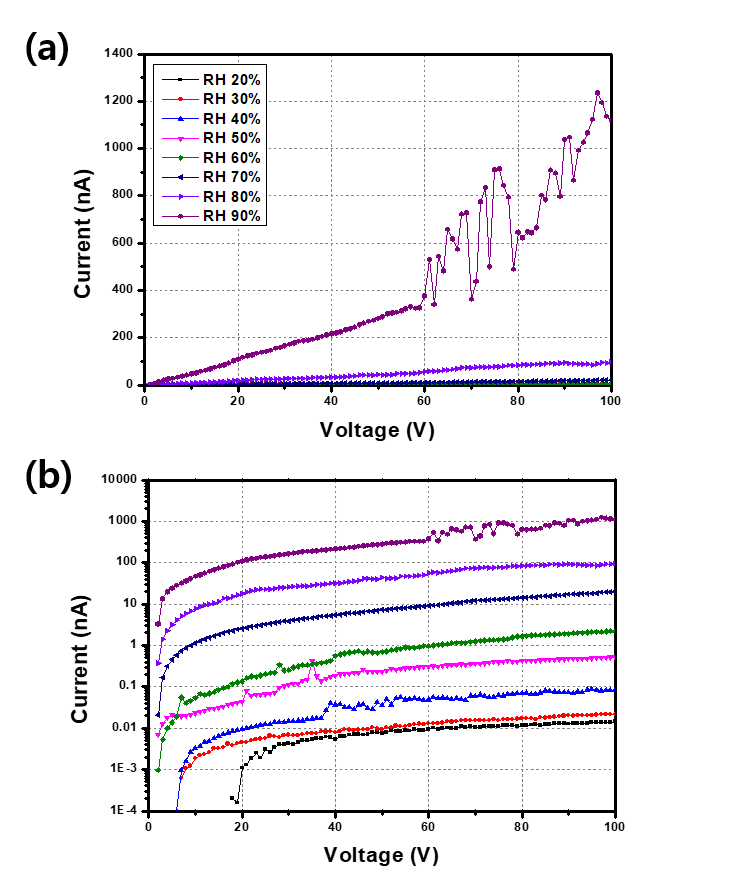


**Figure S26.** (a) *I*–*V* characteristics of the sensor in Remote Mode under varying RH conditions, measured over an applied voltage range of 1–100 V. No breakdown was observed at RH ≤ 80%, whereas a distinct breakdown occurred at 90% RH. (b) Same data as in (a), replotted on a logarithmic scale.


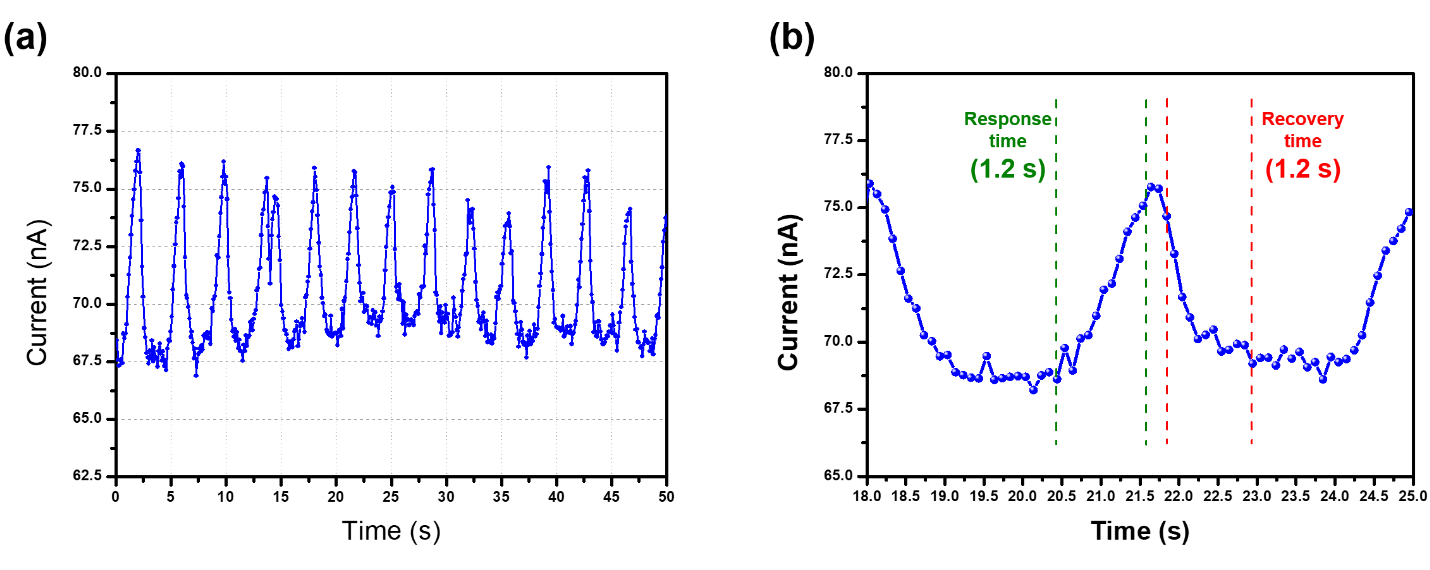


**Figure S27.** (a) Contact Mode respiratory monitoring measurement (normal breathing of 15.9 bpm). (b) Response and recovery times during respiratory monitoring in Contact Mode.

**
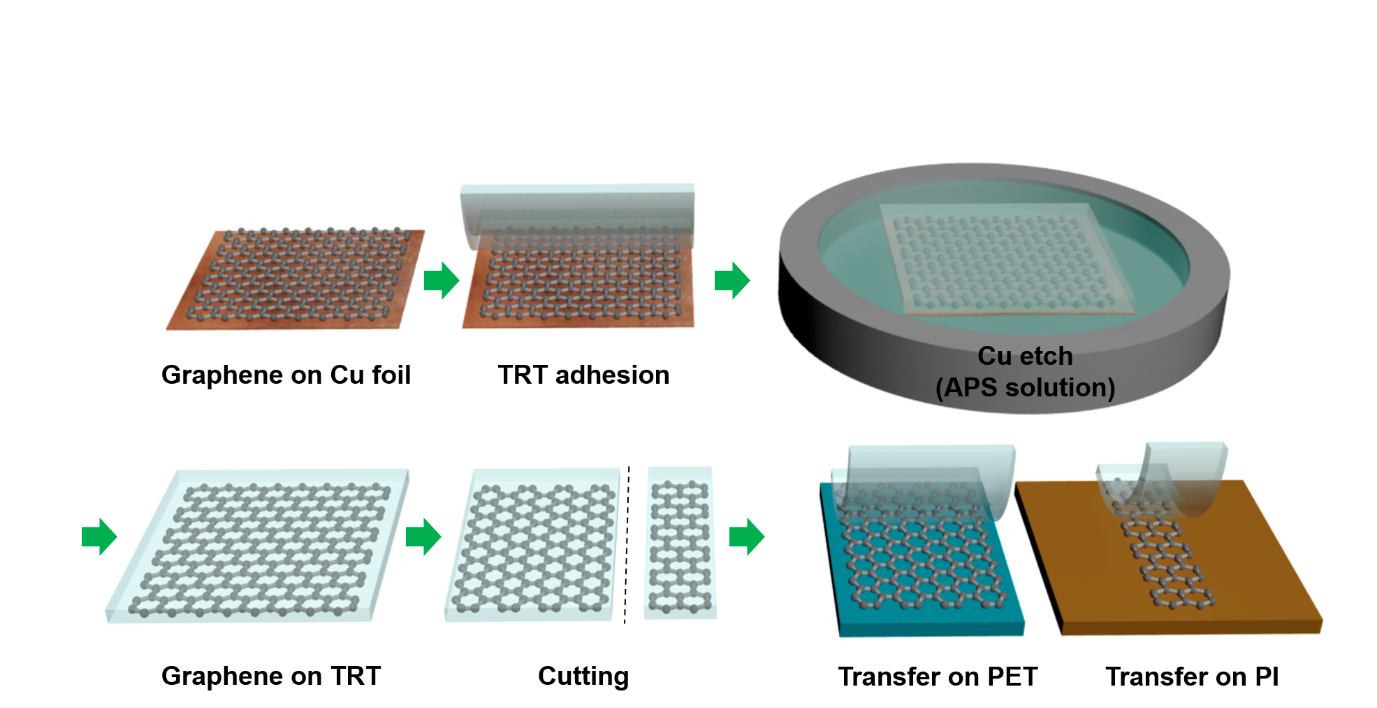
**

**Figure S28.** Schematics of graphene transfer processes onto PI and PET films.


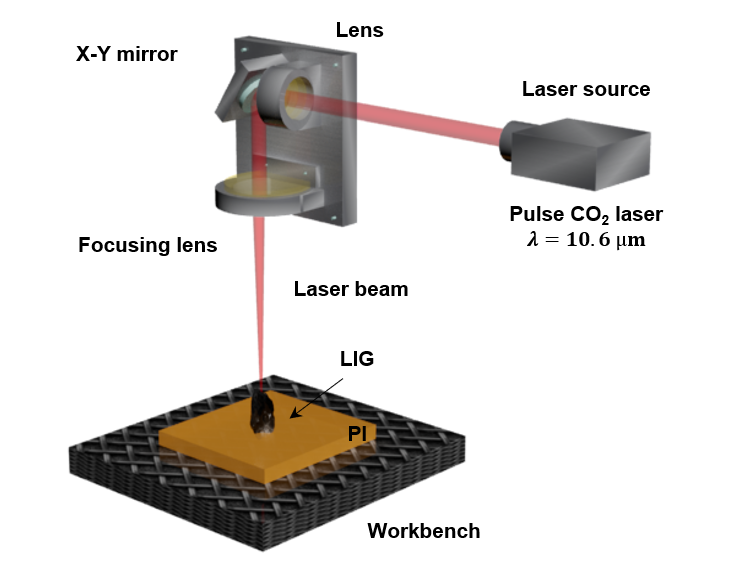


**Figure S29.** Laser specifications (Universal Laser Systems VLS 2.30DT).


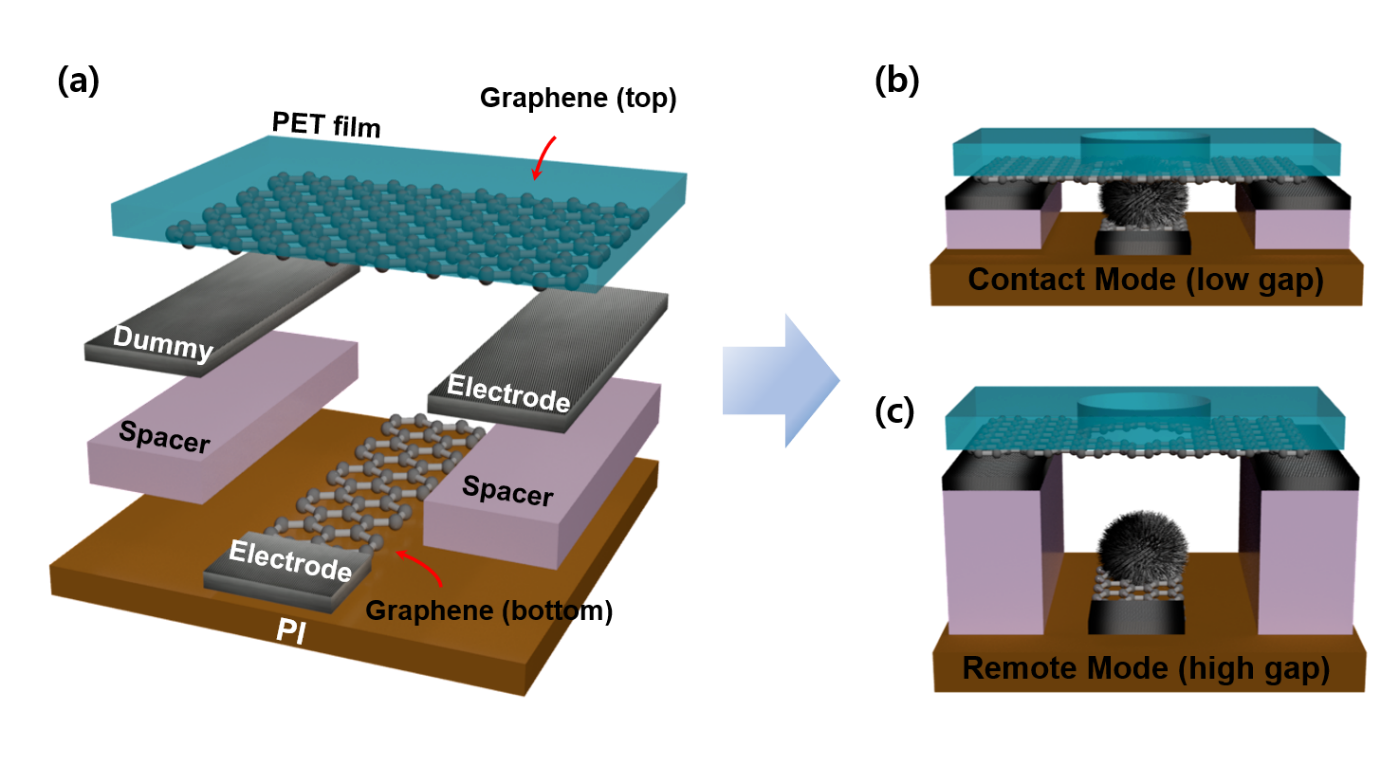


**Figure S30.** Schematics of the (a) components used to fabricate vertical-contact LIG nanotip sensors, (b) Contact Mode sensor, and (c) Remote Mode sensor.


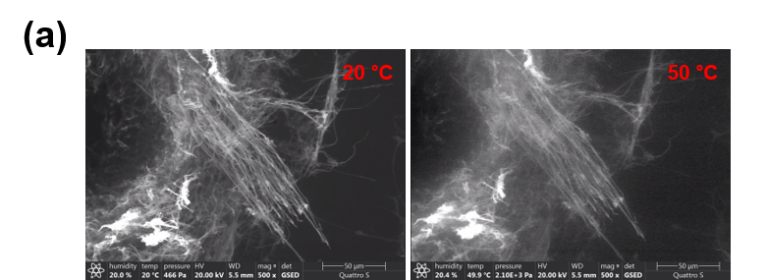


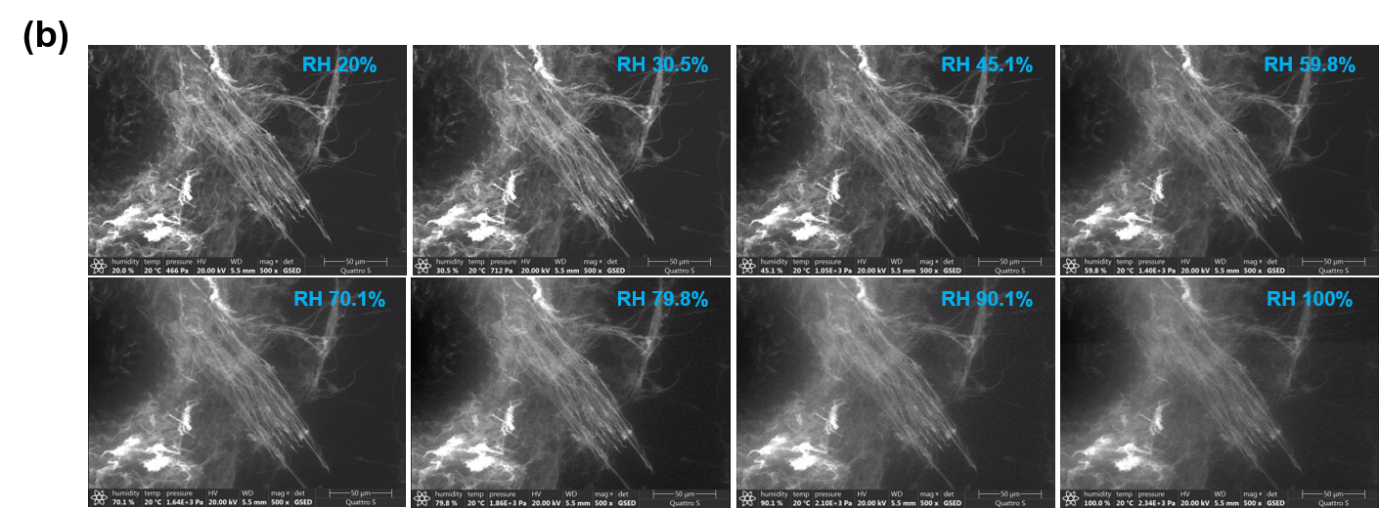


**Figure S31** (a) Environmental SEM (E-SEM) image of the LIG nanotip structure observed at 20 °C and 50 °C under low-humidity conditions, confirming that mild heating does not induce structural deformation or collapse of the LIG architecture. (b) E-SEM images of the same LIG structure under gradually increasing RH from 20% to 100%), demonstrating stable morphology and no irreversible saturation under prolonged exposure to high humidity. These results support the sensor’s reversible water adsorption/desorption behavior and long-term robustness under cyclic humidity conditions.

**References**

[S1] O. I. Tolstikhin, B. Madsen, *Physical Review A*, **2011**, *84*, 053423.

[S2] Y. P. Raizer, J. E. Allen, *Gas Discharge Physics*, Springer **1997**.

[S3] N. Shimoi, S. Tanaka, *Carbon*, **2009**, *47*, 1258, S.-i.

[S4] M. Benziada*, IEEE Transactions on Dielectrics and Electrical Insulation* (*Trans.:* A. Boubakeur, A. Mekhaldi, *IEEE*), **2018**, *25*, 2093.

[S5] L. Ma, A. Varveri, R. Jing, C. Kasbergen, S. Erkens, *Materials and Design*, **2022**, *222*, 111028.

[S6] Z. Zhang, H. Zhu, W. Zhang, Z. Zhang, J. Lu, K. Xu, Y. Liu, V. Saetang, *Carbon*, **2023**, *214*, 118356.

[S7] J. T. Robinson, J. Culbertson, M. Berg, T. Ohta, *Scientific Reports*, **2018**, *8*, 1.

[S8] X. Liu, W. Wu, B. Guo, M. Cui, H. Ma, Z. Zhang, R. Zhang, *Journal of Materials Chemistry C*, **2021**, *9*, 214.
